# Frailty prediction in patients with chronic digestive system diseases: based on multi-task learning model

**DOI:** 10.3389/fmed.2025.1633890

**Published:** 2025-08-26

**Authors:** Sihan Hu, Xiaochuan Guo, Xiaobao Wang, Zixiang Jin, Chenyang Zhou, Lang Tu, Zhoulong Shi, Weiyi Ao, Xin Zhang, Jay Zheng, Xuezhi Zhang, Hui Ye

**Affiliations:** ^1^Department of Integrated Traditional Chinese and Western Medicine, Peking University First Hospital, Institute of Integrated Traditional Chinese and Western Medicine, Peking University, Beijing, China; ^2^Department of Respiratory Diseases, The First Affiliated Hospital of Henan University of Chinese Medicine, Zhengzhou, China; ^3^Hospital of Chengdu University of Traditional Chinese Medicine, School of Clinical Medicine, Chengdu University of Traditional Chinese Medicine, Chengdu, China; ^4^School of Basic Medical Sciences, Chengdu University of Traditional Chinese Medicine, Sichuan, China; ^5^West China School of Public Health/West China Fourth Hospital, Sichuan University, Chengdu, China; ^6^College of the First Clinical Medicine, Chongqing Medical University, Chongqing, China; ^7^Key Laboratory of Carcinogenesis and Translational Research (Ministry of Education/Beijing), Department of Biochemistry and Molecular Biology, Peking University Cancer Hospital and Institute, Beijing, China; ^8^Department of Medical Sciences, Loreal Dermatology Beauty, Paris, France

**Keywords:** chronic digestive system disease, frailty prediction, multi-timepoint prediction, multi-gate mixture-of-experts framework, CHARLS database

## Abstract

**Background:**

Chronic digestive system diseases (CDSD) pose a major health challenge worldwide, significantly increasing morbidity and mortality rates. The frailty index is crucial for assessing patient prognosis. To address the need for proactive healthcare, we developed a multi-timepoint frailty prediction model.

**Methods:**

This study collected data from 565 patients with CDSD, including their frailty assessments at 3 and 6 years of follow-up. Utilizing the Multi-Gate Mixture-of-Experts (MMoE) framework, we built and evaluated five models: Tab Transformer, Convolutional Neural Network (CNN), Deep Neural Network (DNN), Extreme Gradient Boosting (XGBoost) and Random Forest (RF). We comprehensively compared the predictive capabilities of these models on both validation and test sets.

**Results:**

The MMoE framework consistently outperforms single models in predicting both 3-year and 6-year frailty indices across most metrics. Specifically, for 3-year predictions, the single model achieves an accuracy of 0.9801 (95% CI: 0.963–0.990) on the train set and 0.5487 (95% CI: 0.457–0.637) on the test set, while the MMoE model reaches 0.956 (95% CI: 0.933–0.971) and 0.982 (95% CI: 0.938–0.995), respectively. The RF model demonstrated perfect performance, with Micro-AUC values of 1.000 in both training and test sets for both 3-year and 6-year intervals, leading other models in terms of accuracy, precision, recall, F1 score. The Tab Transformer model achieved high Micro-AUC values across all prediction intervals, with values of 0.997 and 0.995 in the training set for 3-year and 6-year predictions, respectively, and corresponding test set values of 0.999 and 0.987.

**Conclusion:**

This MMoE-based approach can predict frailty at key time points, offering insights into frailty progression and aiding clinical decision making. Integrating this AI model into CDSD management can promote early interventions and personalized treatment plans.

## 1 Introduction

Chronic digestive system diseases (CDSD), such as irritable bowel syndrome (IBS), Crohn’s disease, ulcerative colitis, chronic gastritis and cirrhosis, have become important health problems worldwide, affecting the health outcomes of millions of patients (1). In the context of global aging, the traditional medical model based on clinical indicators has been unable to fully cope with the challenges brought by these complex diseases, so more attention needs to be paid to the overall functional status and quality of life of patients (2, 3). Among them, frailty, as a multifactorial age-related clinical syndrome, manifested as a decline in multi-system physiological reserves, is particularly common in patients with CDSD and is receiving increasing attention (2, 4). Since CDSD is often accompanied by nutrient absorption disorders and systemic inflammation, leading to malnutrition and sarcopenia, the process of frailty is accelerated (5, 6). These factors impair muscle strength, energy levels and body elasticity, resulting in patients with CDSD being more susceptible to decreased mobility, fatigue and increased susceptibility to stressors (7). Frailty is closely related to adverse outcomes such as mortality, hospitalization, and complications. Its incidence in patients with CDSD is higher than that in the general population, and it often indicates a poor prognosis (8). Therefore, it is of great clinical significance to assess and predict frailty in patients with CDSD. Frailty is a dynamic state that may improve or worsen over time (9). Studies have shown that frailty is an important indicator for predicting adverse outcomes in patients, such as decreased physical and cognitive functions, increased complications, etc. (10–12). Predicting the future trajectory of frailty evolution based on the patient’s current condition is crucial for preventing hospitalization and premature death.

Traditionally, machine learning (ML) in frailty prediction has relied on individual models such as Random Forest (RF) and Extreme Gradient Boosting (XGBoost), which have demonstrated excellent performance in related domains, for example in chronic obstructive pulmonary disease (COPD) and heart failure cohorts (13, 14). Recent research reveals that while single-model approaches encompassing architectures like the Tab Transformer, Convolutional Neural Network (CNN) (15), Deep Neural Network (DNN), RF, and XGBoost are effective at modeling non-linear and high-dimensional relationships, however, they may fall short when applied in isolation to the heterogeneous and complex clinical data present in the China Health and Retirement Longitudinal Study (CHARLS) database (16–21).

To address these limitations, a Multi-Gate Mixture-of-Experts (MMoE) framework has been proposed as an advanced alternative to traditional single-task models. The MMoE model is an ensemble learning architecture that integrates multiple “expert” models through a gating mechanism, dynamically assigning weights to each expert’s prediction based on the input characteristics (22). The dynamic weighting inherent in MMoE not only enables the model to aggregate complementary information from diverse sources but also ameliorates the risk of overfitting associated with individual models (23). Compared directly with state-of-the-art single-model methods, the MMoE approach exhibits several distinct advantages. While models based on the Tab Transformer, CNN, DNN, RF, and XGBoost have shown high performance on singular predictive outcomes in the CHARLS (24–26), their architectures are inherently rigid—designed to capture isolated patterns rather than the intertwined nature of frailty determinants. In contrast, MMoE’s dynamic gating allows for more nuanced handling of correlations among heterogeneous clinical predictors. This flexibility translates into superior generalization performance when predicting comprehensive frailty indices, as the model effectively leverages both shared and task-specific representations derived from the complex MIMIC data. Moreover, the MMoE architecture employs a shared feature transformation layer that acts as a unified representational subspace for all tasks, also known as soft parameter sharing (27). Therefore, the MMoE framework offers the ability to learn the shared features across different tasks and adjust experts’ weight through task-specific gate networks (28), which deliver a more comprehensive explanation of feature contributions.

This study aims to evaluate and compare the performance of five different expert models—Tab Transformer, CNN, DNN, RF, and XGBoost when integrated via an MMoE framework for predicting the frailty index in patients with CDSD using CHARLS data. To the best of our knowledge, we are the first to apply and compare five different experts for analyzing optimal MMoE architecture performance in frailty prediction. By positioning these cutting-edge models against mainstream approaches, we seek to highlight their advanced capabilities in capturing complex feature interactions, enhancing interpretability, and ultimately improving risk stratification in patient with CDSD populations.

Our contributions can be summarized as follows: We propose the first MMoE-based framework for frailty prediction among 565 patients with chronic disease, utilizing 3 and 6 year follow-up data from the CHARLS; We integrate Tab Transformer, CNN, DNN, RF, and XGBoost as expert models, and demonstrate that the Tab Transformer model and RF model achieve high Micro-AUC performances; We publicly release a reproducible pipeline to support future research on frailty modeling in the context of chronic diseases.

## 2 Materials and methods

**Figure 1:**
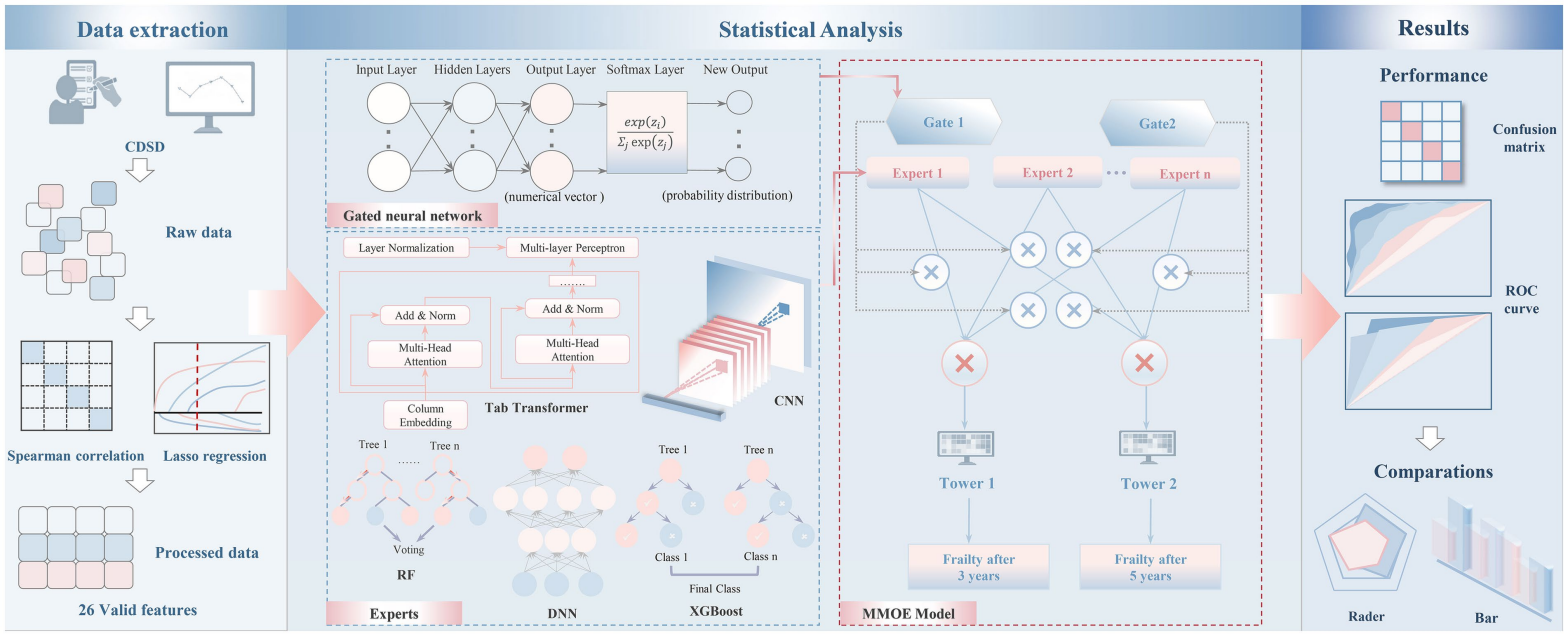
Research map.

This study employed the MMoE framework to predict frailty progression in patients with CDSD using CHARLS data. MMoE, as a multi-task learning model, has shown strong predictive capabilities in medical research and is particularly suitable for handling multi-task scenarios (29, 30). Specifically, each expert network processes input data independently to meet task-specific needs, while the gating network dynamically selects and weights expert outputs. We utilized five models—Tab Transformer, CNN, DNN, RF, and XGBoost—as expert models within this framework to handle multi-objective tasks (Figure 1).

### 2.1 Study population

CHARLS is a nationally representative longitudinal research project investigating population aging through systematic data collection from Chinese households with members aged 45 years or older. The baseline survey conducted in 2011 adopted a multi-stage probability proportional to size sampling strategy, covering 28 provinces with 150 counties, 450 villages, and approximately 10,000 households representing over 17,000 individuals. The survey instrument captures comprehensive information including demographic characteristics, intergenerational financial transfers, health status metrics, healthcare access patterns, employment history, economic status (income, expenditures and assets), supplemented by physical examinations and biological specimen collection. All study procedures received approval from the Biomedical Ethics Committee of Peking University, and researchers may access the anonymized datasets through the official CHARLS website.^1^

### 2.2 Data source and extraction

In this study, we utilized data from three waves of the CHARLS. The baseline survey in 2013 included 17,708 participants, from which we extracted data on demographics, health status, functionality, housing characteristics, healthcare, and insurance information. Focusing on participants diagnosed with CDSD (excluding those with fatty liver, tumors, or cancer) who completed two follow-ups, we extracted relevant information from the 2013 baseline survey (17,708 participants) to predict frailty progression. The 2011 data had too many missing values for our research goal, so we used the 2013 data as the baseline and the 2015/2018 data for frailty labels to maximize the analyzable sample size. Splitting data by region, age, and chronic disease status to ensure proportional representation of subgroups in training/testing sets.

#### 2.2.1 Inclusion criteria

We selected participants diagnosed with CDSD at baseline who also completed at least one follow-up assessments during the study period, including the baseline and one subsequent reassessment. This approach ensured the continuity and reliability of the longitudinal evaluation.

#### 2.2.2 Exclusion criteria

**Figure 2:**
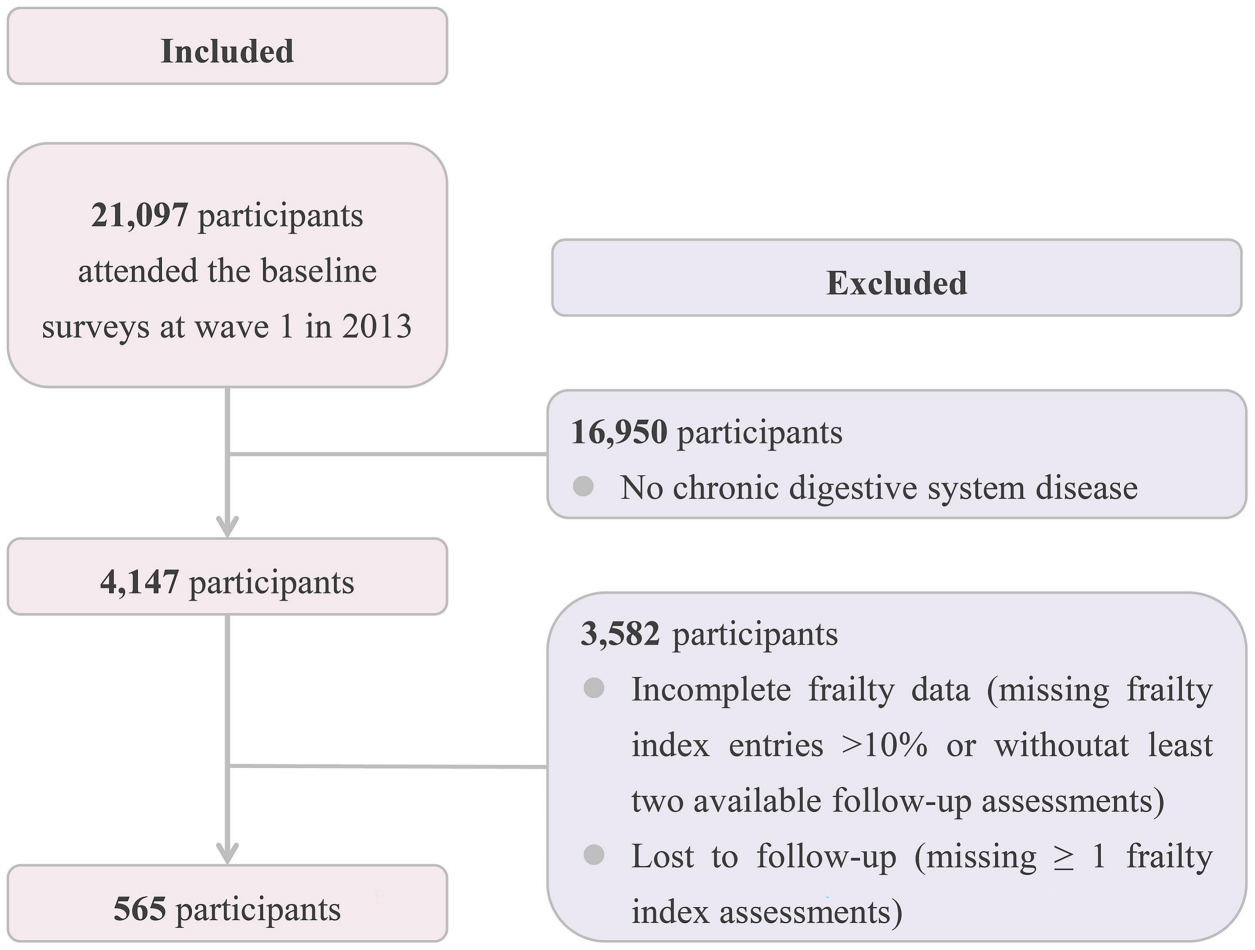
Selection process of the study population.

Participants with more than 10% missing data on frailty index items at baseline were excluded (31). Additionally, individuals who completed fewer than two assessments, including the baseline and at least one follow-up, were excluded. Participants who did not undergo frailty reassessment due to loss to follow-up were also excluded (Figure 2).

### 2.3 Diagnosis of CDSD and frailty

CDSD were identified based on self-reported physician diagnoses, including liver diseases and other digestive system diseases (as defined in Section 2.1 and 2.2). Frailty was assessed using the Frailty Index (FI), which reflects the accumulation of multiple age-related health deficits (32, 33). A total of 32 items were selected to construct the FI, covering a range of conditions such as diseases, symptoms, disabilities, physical functioning, depression, and cognitive function. These items were either dichotomized or treated as a continuous variable to calculate the FI, ranging from 0 to 1, with higher values indicating greater frailty. In line with previous research, frailty status was classified into three categories: robust (FI ≤ 0.10), pre-frail (0.10 < FI < 0.25), and frail (FI ≥ 0.25) (34).

### 2.4 Feature selection

In this study, we extracted 84 predictor variables from baseline data, including demographic characteristics, health status and functionality, healthcare and insurance information, depression (measured using the Center for Epidemiologic Studies Depression Scale, CES-D), and the 2013 frailty index. Frailty data from 2015 and 2018 were used as outcome variables. Predictor variables with over 10% missing data were excluded, while missing values for categorical and continuous variables were imputed using the mode and mean, respectively, to create a complete dataset. Categorical variables were transformed into dummy variables through one-hot encoding to enable their inclusion in the model. Spearman correlation analysis was performed to identify highly correlated features (correlation coefficient > 0.9) (35), with one feature from each pair randomly excluded to minimize multicollinearity (Supplementary formula 1) (36). To further filter a set of features with stronger predictive ability and handle the relationship between features and targets, Least Absolute Shrinkage and Selection Operator (Lasso) regression with L1 regularization was applied, ensuring key predictors were selected while mitigating the risk of overfitting (37). LASSO regression algorithm was conducted on the training set after the dataset was split into training and test sets. Features with coefficients reduced to 0 were excluded, enhancing model sparsity and stability (Supplementary formula 2).

### 2.5 Experimental design

All continuous data were checked for normality using standard tests, and the distribution of variables was carefully assessed before selecting the appropriate statistical test method. Intergroup differences in continuous variables were assessed using the Kruskal-Wallis test and categorical variables were analyzed using the Chi-square test (Supplementary Table 2). All analyses were conducted using Python 3.11, with data preprocessing performed using the “pandas,” “numpy,” and “sklearn” packages. Missing values were addressed through imputation, and the data were split into training and testing sets in an 8:2 ratio, following the current mainstream method (38–40) and using frailty levels from 2015 and 2018 as the outcome variables. The MMoE framework was implemented using the “torch” package to construct predictive models. Within this framework, five algorithms were utilized as expert models: Tab Transformer (Supplementary formula 3) (41), CNN (Supplementary formula 4) (42), DNN (Supplementary formula 5) (43), RF (Supplementary formula 6) (44) and XGBoost (Supplementary formula 7) (45). A Multilayer Perceptron (MLP) (Supplementary formula 8) served as the expert model, synthesizing the outputs from these feature extractors to handle multi-target tasks. The MMoE framework (Supplementary formula 9) (27) enables the sharing of expert networks across tasks while maintaining task-specific learning through independent gate networks. The expert networks process input features to generate task-independent representations, while the gate networks manage the flow of information from each expert to each task (46), dynamically selecting and combining expert outputs based on task requirements. Hyperparameter optimization framework for the expert models within the MMoE architecture was performed using the Optuna (47), and model stability was assessed with 10-fold cross-validation using the “KFold” package. Once optimal hyperparameters were identified, the models were retrained on the entire training set. Performance on the testing set was evaluated using metrics such as accuracy, Micro-AUC, precision, F1 score, recall, and the confusion matrix, with classification reports generated by sklearn. The model with the best performance was selected to forecast frailty progression at future time points in patients with CDSD.

## 3 Results

### 3.1 Baseline characteristics

**Figure 3:**
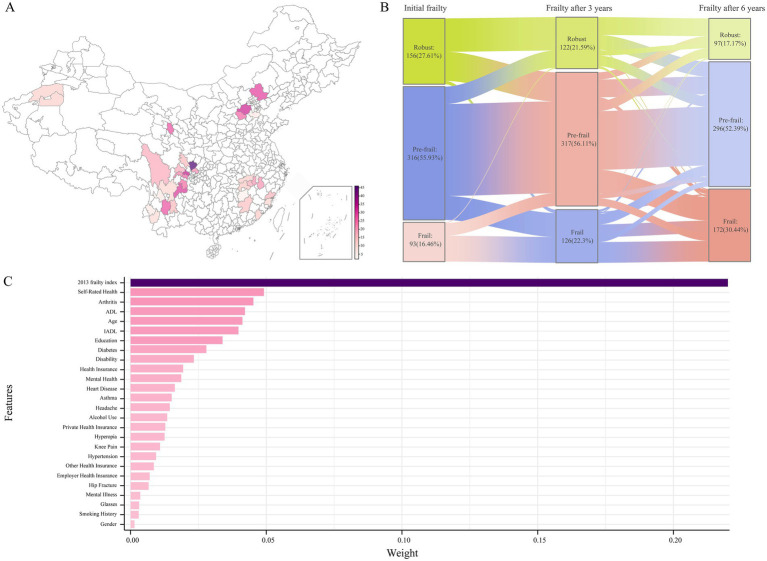
Regional distribution and annual frailty transition of patients. **(A)** Regional distribution of frailty cases in China. **(B)** Yearly transitions in frailty status: robust, pre-frail, frail. **(C)** Feature importance for frailty prediction based on Lasso regression.

This study included 565 adults with CDSD from 8 provinces or municipalities across China, comprising 1 directly controlled municipality and 34 prefecture-level cities (Figure 3A). In 2015, the cohort consisted of 122 (21.59%) robust patients, 317 (56.11%) pre-frail patients, and 126 (22.30%) frail patients. In 2018, the distribution had shifted, with 97 (17.17%) robust patients, 296 (52.39%) pre-frail patients, and 172 (30.44%) frail patients.

### 3.2 Feature selection

A total of 84 independent variables, encompassing both categorical and continuous data, were analyzed. Spearman correlation analysis was performed on these features, revealing that 7 pairs had correlation coefficients greater than 0.9. Based on the frequency of highly correlated features, 7 features were removed to mitigate multicollinearity. Subsequent Lasso regression analysis was conducted, during which feature coefficients decreased progressively as the Lambda value increased, indicating a higher degree of regularization. An optimal balance between model complexity and generalization ability was achieved at a Lambda value of 0.022. At this point, Lasso regression penalized less relevant features, resulting in 26 features with coefficients reduced to 0, which were subsequently excluded from the model.

To assess the significance of the relationships between the retained independent variables and the dependent variable, a polynomial equation test was conducted, confirming the validity of 26 features. Among these, the five features with the highest weights were Initial Frailty Index (0.220), Self-Rated Health (0.049), Arthritis (0.045), Activities of Daily Living (ADL) score (0.042), and Age (0.041) (Figure 3C and Supplementary Table 1). Based on the selected features, the baseline demographic and clinical characteristics of the participants were listed as follows: Initial Frailty Index, Self Rated Health, Arthritis, ADL, Age, IADL, Education, Diabetes, Disability, Medical Insurance Ownership, Mental Health, Hearte, Asthmae, Headache, Drink, Private Medical Insurance, Vision, Knee Pain, Hypertension, Other Medical Insurance, Urban Employee Medical Insurance, Hip Fracture, Mental Illness, Glass, Smoke, Gender (Supplementary Table 2).

### 3.3 Parameter selection

After feature selection, to build an efficient multi-task learning architecture, we trained models within the MMoE framework using the Pytorch package and optimized the parameters for five expert models: Tab Transformer, CNN, DNN, RF and XGBoost. Through hyperparameter optimization and 10-fold cross-validation, the optimal parameter combinations were determined to ensure better performance of the expert models within the multi-task framework.

To optimize the performance of the expert models within the MMoE framework, hyperparameter optimization framework was conducted using the Optuna to ensure the best possible predictive outcomes.

For the Tab Transformer, the optimal parameters were set with a dimensionality of 32 for input features and a depth of 6 for the transformer layers. The model utilized 4 attention heads, each with a dimension of 32, and the hidden layers multiplication was set to 4. The number of experts was configured to 3. These settings enable the model to capture complex interactions in tabular data, particularly in datasets with mixed feature types.

In the case of the CNN, the model was fine-tuned with 64 output dimensions after the convolutional layers and 256 for the fully connected layer output. A batch size of 32 and a learning rate of 0.001 were chosen to ensure efficient training. The number of experts was set to 3 to balance feature extraction and model efficiency. These configurations were aimed at enabling the CNN to effectively extract hierarchical features from input data, improving predictive performance.

The DNN expert model was optimized with a hidden dimension of 256 and a batch size of 64. The learning rate was set to 0.001, and the number of experts was set to 3. These hyperparameters were selected to model complex, non-linear relationships within the data while enhancing the model’s predictive capabilities.

The RF model was configured with 200 estimators and a maximum tree depth of 5. The minimum samples required to split a node were set to 5, and the minimum samples required at a leaf node were set to 4. The number of features considered for splitting was limited to the square root of the total features, and bootstrapping was turned off.

For the XGBoost model, the optimal hyperparameters were selected as follows: the number of experts was set to 3, with 100 estimators, a learning rate of 0.01, and a maximum tree depth of 5. Additionally, the minimum child weight was set to 5, with gamma set to 0. The subsample rate and column sample by tree were both set at 0.8, while L1 regularization was set to 0 and L2 regularization to 1. These configurations were chosen to balance model complexity and prevent overfitting, ensuring better generalization performance.

### 3.4 Construction and validation of a frailty prediction model for patients with CDSD

The MMoE framework consistently outperforms single models in predicting both 3-year and 6-year predictions across most evaluated metrics. For the 3-year and 6-year predictions, the MMoE model shows a significant improvement in accuracy and Micro-AUC compared to the single model. While the Tab Transformer’s single model achieves an accuracy of 0.999 on the train set and 0.549 on the test set, the MMoE model reaches 0.997 and 0.999 for the 3-year with Tab Transformer’s experts, respectively. Similar trends are observed in the other four models (Supplementary Figures 1–16 and Supplementary Tables 3, 4).

**Figure 4:**
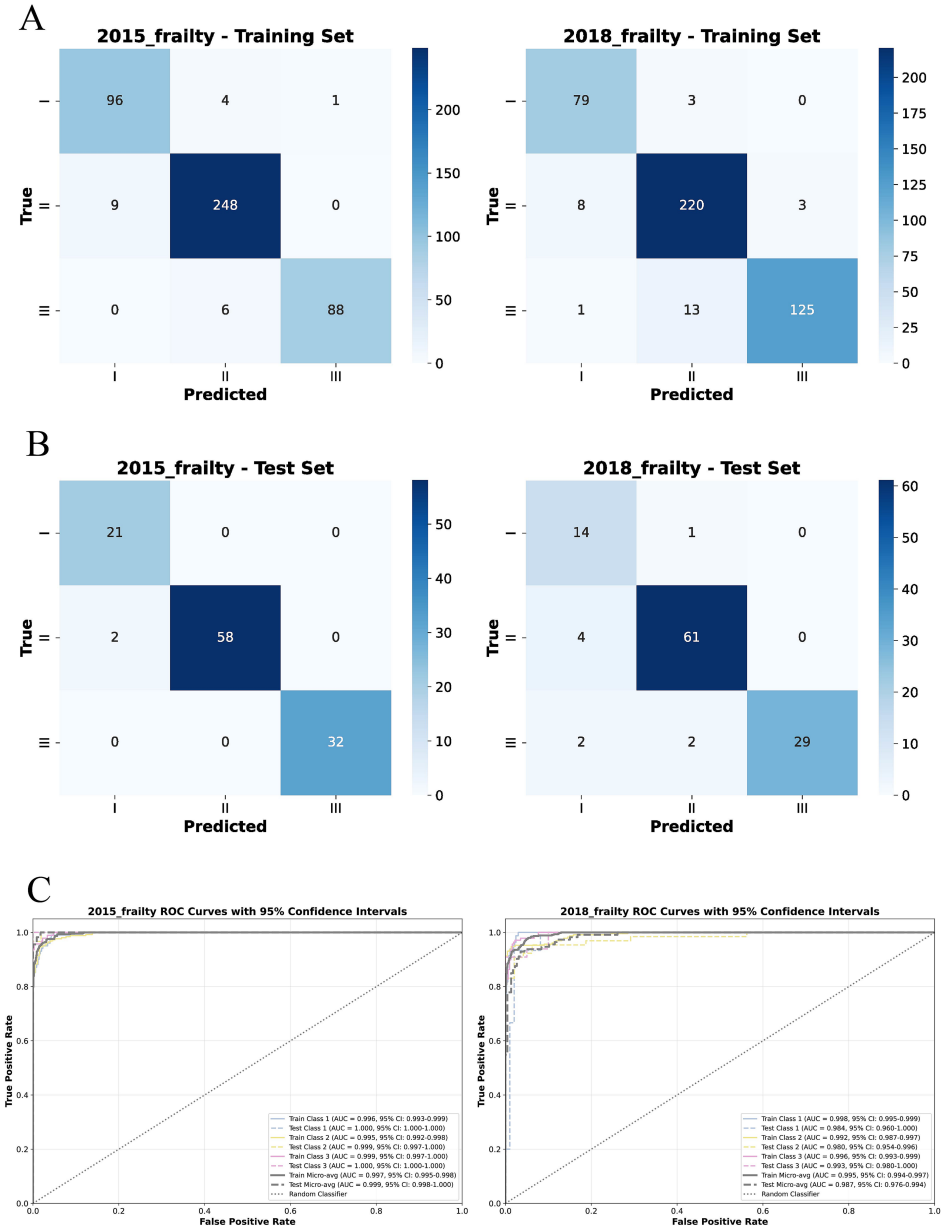
Confusion matrices and ROC curves of the MMoE model using tab transformer’s experts for frailty prediction at 3 and 6 Years. **(A)** Confusion matrices for the training set at 3-year and 6-year intervals, showing predictions for Robust, Pre-frail, and Frail categories. **(B)** Confusion matrices for the test set at the corresponding intervals. **(C)** ROC (Receiver Operating Characteristic Curve) curves for each prediction interval, with ROC values indicating the model’s discriminatory ability between classes (AUC, area under the curve; CI, Confidence interval; Micro-avg, Micro-averaging; ROC, Receiver Operating Characteristic Curve).

**Figure 5:**
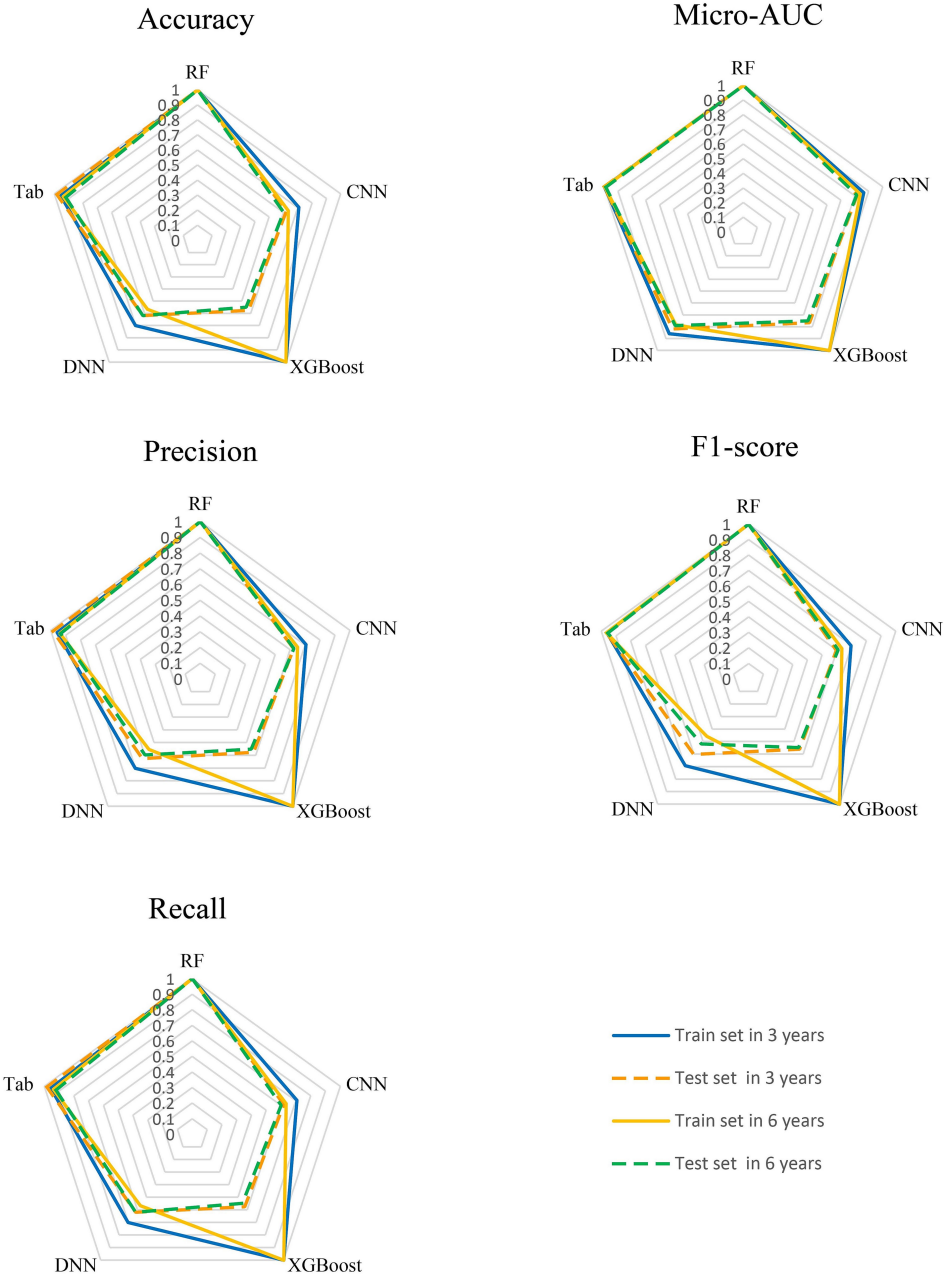
Comparative performance of MMoE models for frailty index prediction across multiple metrics at 3-years and 6-years: Accuracy, Micro-AUC, Precision, F1 Score and Recall. Each radar plot displays the performance of the models on both training (solid lines) and test sets (dashed lines), with colors representing the time intervals: blue for train set in 3 years, orange for test set in 3 years, yellow for train set in 6 years and green for test set in 6 years. Models positioned closer to the outer edge indicate stronger performance on the respective metric.

The Tab Transformer model achieved the high Micro-ROC values across all prediction intervals. Micro-ROC values of 0.997 and 0.995 in the training set for the 3-year and 6-year predictions. In the test, the corresponding Micro-AUC values were 0.999 and 0.987 (Figures 4, 5).

The CNN model also demonstrated strong performance, with Micro-AUC values of 0.865 and 0.839 in the training set for the 3-year and 6-year predictions. In the test set, the Micro-AUC values were 0.817 and 0.814 (Tables 1, 2), showing a more noticeable decrease in discriminatory power over time. This trend suggests that while the model performs excellently on the training data, the performance diminishes as the prediction horizon lengthens, particularly in the test set (Figure 5 and Supplementary Figure 1).

**Table 1:** Performance metrics of MMoE models for 3-year frailty index prediction.

Model	Dataset	Accuracy (95%CI)	Micro-AUC	Precision	F1-score	Recall
Tab Transformer	Train set	0.956 (0.933–0.971)	0.997	0.957	0.956	0.956
	Test set	0.982 (0.938–0.995)	0.999	0.984	0.983	0.982
CNN	Train set	0.708 (0.664–0.748)	0.865	0.707	0.697	0.708
	Test set	0.620 (0.527–0.704)	0.817	0.623	0.596	0.620
DNN	Train set	0.701 (0.658–0.742)	0.859	0.702	0.693	0.701
	Test set	0.620 (0.527–0.704)	0.820	0.625	0.603	0.620
RF	Train set	1.000 (0.992–1.000)	1.000	1.000	1.000	1.000
	Test set	1.000 (0.992–1.000)	1.000	1.000	1.000	1.000
XGBoost	Train set	1.000 (0.992–1.000)	1.000	1.000	1.000	1.000
	Test set	0.575 (0.483–0.662)	0.767	0.576	0.562	0.575

**Table 2:** Performance metrics of MMoE models for 6-year frailty index prediction.

Model	Dataset	Accuracy (95%CI)	Micro-AUC	Precision	F1-score	Recall
Tab Transformer	Train set	0.938 (0.911–0.957)	0.995	0.940	0.938	0.938
	Test set	0.920 (0.856–0.958)	0.987	0.933	0.923	0.920
CNN	Train set	0.635 (0.590–0.678)	0.839	0.651	0.634	0.635
	Test set	0.602 (0.510–0.687)	0.814	0.626	0.609	0.602
DNN	Train set	0.566 (0.520–0.611)	0.784	0.555	0.459	0.566
	Test set	0.620 (0.527–0.704)	0.790	0.598	0.518	0.620
RF	Train set	1.000 (0.992–1.000)	1.000	1.000	1.000	1.000
	Test set	1.000 (0.992–1.000)	1.000	1.000	1.000	1.000
XGBoost	Train set	1.000 (0.992–1.000)	1.000	1.000	1.000	1.000
	Test set	0.549 (0.457–0.637)	0.751	0.551	0.549	0.549

For the DNN model, the Micro-AUC values in the training set were consistently high, recorded as 0.859 and 0.784 for the 3-year, 6-year, for all prediction intervals. On the test set, the Micro-AUC values were slightly lower, recorded as 0.820 and 0.790 for the 3-year and 6-year intervals (Tables 1, 2). This pattern highlights that although the DNN model maintains high predictive capability in the training set, there is a modest decline in performance when tested on unseen data, particularly for longer-term predictions (Figure 5 and Supplementary Figure 2).

For the RF model, Micro-AUC values in the training set were high at 1.000 and 1.000 for the 3-years and 6-years intervals. The test set Micro-AUC values were 1.000 and 1.000 (Tables 1, 2). This indicates that the RF model was able to perfectly distinguish between different frailty classes in the training data, and more importantly, maintained this discriminative ability when applied to unseen test data (Figure 5 and Supplementary Figure 2).

For the XGBoost model, Micro-AUC values in the training set were high at 1.000 and 1.000 for the 3-years and 6-years intervals. The test set Micro-AUC values were 0.767 and 0.751 (Tables 1, 2), showing a noticeable decrease over the longer-term predictions. This suggests that while XGBoost performs well in both training and test sets, there is a decline in predictive discrimination as the interval extends, particularly in the test data (Supplementary Figures 4, 5).

## 4 Discussion

Frailty is a growing global health burden driven by aging populations and an increasing prevalence of chronic diseases (4, 48, 49). Severe frailty often leads to multi-organ functional decline, and higher healthcare costs, placing significant physical and mental strain on patients (50, 51). Among patients with CDSD, frailty is particularly common and strongly associated with prognosis. Despite its clinical importance, the development of predictive tools tailored specifically to patients with CDSD remains inadequate, hindering the timely identification and effective management of high-risk individuals. Recent studies have underscored the potential of automated prediction models to bridge this gap in frailty assessment and intervention (52). For instance, Wu et al. (53) utilized interpretable machine learning to evaluate frailty trajectories in older adults, highlighting the growing role of advanced analytics in geriatric care. However, most existing models are designed for general populations or specific conditions, leaving patients with CDSD underrepresented. Multi-timepoint prediction models, which forecast frailty status at several future intervals, offer distinct advantages by capturing the progression of frailty over time. This enables clinicians to anticipate changes, personalize interventions, and evaluate the long-term impact of treatments (54–56). Addressing these needs, this study employed a multi-task learning framework to develop a predictive algorithm based on 26 clinical variables. The model aims to forecast frailty status in patients with CDSD at 3 and 6 years into the future. Among the expert models trained, the Tab Transformer model demonstrated superior performance, achieving the highest accuracy and Micro-AUC across all time points. By focusing on CDSD-specific outcomes, this study contributes to closing the gap in predictive tools for this vulnerable patient population.

Five features with the highest weights (Initial Frailty Index, Self-Rated Health, Arthritis, ADL, Age) hold significant clinical implications for predicting frailty. The Initial Frailty Index quantifies accumulated health deficits, serving as a robust marker of vulnerability and predictor of adverse outcomes. Self-Rated Health captures subjective well-being, providing insights into potential declines not yet evident through objective measures (57). Arthritis highlights the impact of chronic conditions on functional decline, while ADL performance reflects independence and signals frailty-related functional deterioration (58, 59) Age, a fundamental risk factor, interacts with these clinical indicators to explain heterogeneity in frailty profiles. Together, integrating these predictors into clinical practice facilitates early identification of individuals at high risk for frailty and its complications and guide clinicians in designing tailored plans addressing modifiable factors.

This research is based on patients with CDSD from the CHARLS data. Using demographic characteristics, health status and functionality, biomarkers, healthcare and insurance information, and the Center for Epidemiologic Studies Depression Scale (CES-D10) from 2013 as features, we predicted frailty status in 2015 and 2018 (i.e., 3 and 6 years later) as outcome variables. Feature selection was conducted through Spearman correlation tests, Lasso regression analysis. Subsequently, based on the MMoE framework, we utilized Tab Transformer, CNN, DNN, RF and XGBoost as expert models, selecting the optimal model to construct a double time-point frailty prediction model for patients with CDSD. This model provides a reference for predicting frailty levels and enabling early intervention in clinical settings for these patients.

Additionally, during feature selection, we found that the MMoE framework offers significant advantages. First, by sharing a base network and multiple expert networks, it effectively captures the commonalities and differences between tasks, improving the overall generalization ability of the model (60). Second, the MMoE framework reduces interference between tasks, allowing each task’s specific objectives to be learned independently through different gating mechanisms, thereby avoiding overfitting to any single task. Multi-task learning also improves efficiency through information sharing, particularly in cases where data is sparse or imbalanced across tasks. The strength of the Tab Transformer model lies in its ability to capture complex, high-order interactions between features in tabular data through a transformer-based architecture (41). By utilizing multi-head attention mechanisms and layer normalization, Tab Transformer is able to effectively model the dependencies between input features while maintaining computational efficiency, which leverage self-attention mechanisms to effectively capture feature interactions in tabular data, outperform conventional techniques in handling heterogeneous clinical datasets (61). Flexible in handling both categorical and continuous variables through embeddings, it scales well with high-dimensional data. In frailty prediction, Tab Transformer dynamically weights feature relevance over time, adapting to evolving disease patterns. This adaptability, combined with strong feature extraction and generalization, enables robust performance across training and testing sets, making it well-suited for accurate, time-sensitive frailty prediction and personalized clinical decision-making (52, 62). RF model’s superior performance in our study can be attributed to its robustness, ability to handle complex data, interpretability, and resistance to overfitting. It excels at capturing non-linear relationships and interactions between features without requiring explicit feature engineering, making them well-suited for this type of predictive task (63, 64). These advantages make it a powerful tool for frailty prediction and highlight its potential for clinical application in identifying high-risk individuals and guiding personalized interventions.

This study has several limitations. Although we strictly controlled the proportion of missing values in the CHARLS features (10%), potential impacts on model training remain. Additionally, due to the black-box nature of deep learning models and the complexity of the MMoE framework, the model’s ability to interpret variables is limited, which affects its transparency (65, 66). Finally, although the CHARLS database includes populations from various regions across China, it is based solely on Chinese data, limiting the model’s generalizability. We explicitly acknowledge that CHARLS’ sampling framework—while nationally representative—still underrepresents affluent urban populations and may limit extrapolation to cohorts with divergent characteristics. Future work should validate these findings using multi-center cohorts to ensure broader applicability. In forthcoming research, we plan to conduct analyses on diseases relevant to our investigative focus, thereby enriching the frailty prediction model system for multiple disease types.

## 5 Conclusion

This study makes three principal contributions. First, we introduce the first multi-timepoint frailty-prediction framework tailored to CDSD patients, fusing five heterogeneous expert models—Tab Transformer, CNN, DNN, RF and XGBoost—within an MMoE architecture that dynamically weights task-specific gates. Second, the ensemble yields state-of-the-art performance: RF attains perfect discrimination (Micro-AUC = 1.000), while Tab Transformer sustains exceptional generalizability (3-year test AUC = 0.982; 6-year test AUC = 0.920). Third, we identify actionable drivers—initial frailty index, self-rated health, and ADL score—clinically validated through interpretation.

Moving forward, we propose two avenues: (1) validate the framework in multi-center, multi-ethnic cohorts to broaden external validity; (2) incorporate serial laboratory and imaging biomarkers to capture temporal dynamics and further refine time-sensitive predictions. Together, these steps will advance precision geriatrics for CDSD patients.

## Data Availability

Publicly available datasets were analyzed in this study. This data can be found at: https://charls.pku.edu.cn/.

## Footnotes

^1^ http://charls.pku.edu.cn
