# Supplementary material for: Frailty prediction in patients with chronic digestive system diseases: based on multi-task learning model
Source: Front Med (Lausanne). 2025 Aug 26;12:1633890. doi: 10.3389/fmed.2025.1633890 (PMC12417108; doi:10.3389/fmed.2025.1633890)
Supplement: Supplementary file 1 [file Data_Sheet_1.docx]

**Supplementary materials**

**Formula 1：**

$$\rho=\frac{1-\left[ 6*\Sigma\left( d^{2} \right) \right]}{n*\left( n^{2}-1 \right)}$$

The symbol *ρ* represents the Spearman correlation coefficient, *d* denotes the difference between the ranks of each pair of variables, and *n* refers to the sample size.

**Formula 2：**

$$\hat{\beta}=\underset{\beta}{argmin} \left( {\frac{1}{2N}\sum_{i=1}^{N} \left( y_{i}-\left( \beta_{0}+\sum_{j=1}^{p} \beta_{j}X_{ij} \right) \right)}^{2}+\lambda\sum_{j=1}^{p} |\beta_{j}| \right)$$

*N* represents the number of observations, and *y_i_* is the actual value for observation *i*. *X_ij_* denotes the value of predictor *j* for observation *i*, while *β_0_* is the intercept term, and *β_j_* represents the coefficient for predictor *j*. The parameter *λ*, also referred to as alpha, is the regularization parameter that controls the degree of shrinkage applied to the coefficients. The L1 norm $\sum_{j=1}^{p} |\beta_{j}|$ is used in Lasso to penalize the absolute values of the regression coefficients.

**Formula 3：Tab Transformer**

$$h=MLP\left( concat\left( \text{Transformer}\left( E \right),x^{\left( n \right)} \right) \right)$$

*(1)*

$$E=[e_{1},e_{2},\ldots,e_{k}],e_{i}=W_{i}[x_{i}]$$

*(2)*

$h$ is the output prediction after processing both categorical and numerical features. $MLP$ is a multi-layer perceptron applied for prediction tasks.$concat$ is the Concatenation operation to combine transformed categorical features with numerical features.Transformer(E) is The categorical feature embeddings E processed by the Transformer encoder.E is a Categorical feature embeddings, where each feature is embedded into a continuous space.x(n) are numerical features directly added to the concatenated input.$x_{i}$ is the categorical feature value for feature $i$.$W_{i}$ is the embedding matrix for feature $i$. $k$ is the number of categorical features.

**Formula 4：CNN**

$$h=\mathrm{Softmax}\left( W^{(o)}\cdot\mathrm{Flatten}\left( \mathrm{Pool}\left( \mathrm{ReLU}\left( \mathrm{Conv}\left( x,W^{(c)} \right) \right) \right) \right)+b^{(o)} \right)$$

$h$ is the final output.$x$ is input data $W^{(c)}$ is learnable weights (kernels) of the convolutional layer$.\mathrm{Conv}\left( x,W^{(c)} \right)$is convolution operation that extracts feature maps $\mathrm{ReLU}(z)=max(0，z$）is Rectified Linear Unit activation function to introduce non-

linearity.$\mathrm{Pool}\left( \cdot\right)$ is a pooling operation.$\mathrm{Flatten}\left( \cdot\right)$ is a flattening operation to convert feature maps into a vector for fully connected layers.$W^{(o)},b^{(o)}$ are weights and biases of the output layer.$\mathrm{Softmax}\left( \cdot\right)$is an activation function to convert logits into probabilities.

**Formula 5：DNN**

$$h=\sigma\left( W^{(L)}\cdot\sigma\left( W^{(L-1)}\cdot\sigma\left( \ldots\sigma\left( W^{(1)}\cdot x+b^{(1)} \right)+b^{(L-1)} \right) \right)+b^{(L)} \right)$$

$h$ is the final output of the network $x$ is Input data.$W^{(l)}$ is weight matrix of layer $l$=1,2...$L$）$b^{(l)}$ is the Bias vector for layer $l$ $\sigma(\cdot)$ activation function applied element-wise to introducenon-linearity.$L$ is the total number of layers in the network.

**Formula 6：RF**

$$\hat{y}_{i}=\frac{1}{K}\sum_{k=1}^{K} f_{k}(x_{i})$$

$\hat{y}_{i}$ is the predicted value for the $i$-th observation. $x_{i}$ is the input features for the $i$-th observation. $f_{k}(x_{i})$ is the prediction from the $k$-th decision tree. $K$ is the total number of trees in the random forest.

**Formula 7：XGBoost**

$$\hat{y}_{i}=\sum_{k=1}^{K} f_{k}(x_{i}),f_{k}\mathcal{\in F}$$

$\hat{y}_{i}$ is the predicted value for the $i$-th observation.$x_{i}$ is the input features for the $i$-th observation.$f_{k}(x_{i})$ is the $k$-th decision tree in the ensemble. $K$ is the total number of trees. $\mathcal{F}$ is space of regression trees.

**Formula 8：MLP**

$$\boldsymbol{h}_{\boldsymbol{j}}^{\left( \boldsymbol{l} \right)}\boldsymbol{=f}\left( \sum_{\boldsymbol{i=1}}^{\boldsymbol{n}^{\left( \boldsymbol{l-1} \right)}} \boldsymbol{w}_{\boldsymbol{ij}}^{\left( \boldsymbol{l} \right)}\boldsymbol{h}_{\boldsymbol{i}}^{\left( \boldsymbol{l-1} \right)}\boldsymbol{+}\boldsymbol{b}_{\boldsymbol{j}}^{\left( \boldsymbol{l} \right)} \right)$$

$h_{j}^{\left( l \right)}$ is the output of the *j-th* neuron in layer *l*. It is calculated by applying an activation function $h_{i}^{\left( l-1 \right)}$is the output of the *i*-th neuron in the previous layer. $w_{ij}^{\left( l \right)}$is the weight connecting the iii-th neuron in the previous layer to the *j*-th neuron in layer *l*. $b_{j}^{\left( l \right)}$is the bias of the *j-th* neuron in layer *l*. $n^{\left( l-1 \right)}$is the number of neurons in the previous layer *f* (·): Common activation functions.

**Formula 9: MMoE**

$$\boldsymbol{e}_{\boldsymbol{i}}\boldsymbol{=}\boldsymbol{f}_{\boldsymbol{i}}\left( \boldsymbol{x;}\boldsymbol{\theta}_{\boldsymbol{i}} \right)\boldsymbol{,}\quad\boldsymbol{i=1,2,}\boldsymbol{\ldots}\boldsymbol{,E}$$

(1)

$$\boldsymbol{g}_{\boldsymbol{t,i}}\boldsymbol{=}\frac{\mathbf{ex}\mathbf{p} \left( \boldsymbol{w}_{\boldsymbol{t,i}}^{\boldsymbol{\top}}\boldsymbol{x} \right)}{\sum_{\boldsymbol{j=1}}^{\boldsymbol{E}} \mathbf{ex}\mathbf{p} \left( \boldsymbol{w}_{\boldsymbol{t,j}}^{\boldsymbol{\top}}\boldsymbol{x} \right)}\boldsymbol{,}\quad\boldsymbol{i=1,2,}\boldsymbol{\ldots}\boldsymbol{,E}$$

(2)

$$\boldsymbol{o}_{\boldsymbol{t}}\boldsymbol{=}\sum_{\boldsymbol{i=1}}^{\boldsymbol{E}} \boldsymbol{g}_{\boldsymbol{t,i}}\boldsymbol{\cdot}\boldsymbol{e}_{\boldsymbol{i}}$$

(3)

$e_{i}$ is the output of the *i*−th expert network. $f_{i}\left( . \right)$ is the A neural network representing the *i*−th expert. $\theta_{i}$ is the parameters of the *i*−th expert. $E$ is the total number of expert networks. $g_{t,i}$ is the weight of expert *i* for task t, assigned by the gating network. $w_{t,i}^{T}$ is the parameters of the gating network for task t. *x* is the shared input feature vector. $o_{t}$ is the shared representation for task t, aggregated using the gating weights. $g_{t,i}$ is the Gating network weight for expert *i* and task *t*. $e_{i}$ is the Output of expert *i*.

**Supplementary Table 1** Feature Importance from Lasso Regression Analysis

| **Feature** | **Importance** |
| --- | --- |
| Initial Frailty Index | 0.220072051 |
| Self Rated Health | 0.049147749 |
| Arthritis | 0.045242303 |
| ADL | 0.042133290 |
| Age | 0.041198594 |
| IADL | 0.039740059 |
| Education | 0.033872527 |
| Diabetes | 0.027919920 |
| Disability | 0.023319508 |
| Medical Insurance Ownership | 0.019362542 |
| Mental Health | 0.018669526 |
| Hearte | 0.016278558 |
| Asthmae | 0.015137947 |
| Headache | 0.014446058 |
| Drink | 0.013468137 |
| Private Medical Insurance | 0.012783202 |
| Vision | 0.012490381 |
| Knee Pain | 0.010806270 |
| Hypertension | 0.009332143 |
| Other Medical Insurance | 0.008512885 |
| Urban Employee Medical Insurance | 0.006990322 |
| Hip Fracture | 0.006604770 |
| Mental Illness | 0.003521611 |
| Glass | 0.003096585 |
| Smoke | 0.002946618 |
| Gender | 0.001392891 |

**Supplementary Table 2** Statistical tests of Baseline Characteristics

| **Variable** | **Variable type** | **Overall description** | **Group 0 (N=97)** | **Group 1 (N=296)** | **Group 2 (N=172)** | ***P*-value** | **Test method** |
| --- | --- | --- | --- | --- | --- | --- | --- |
| age | Continuous Variable | 59.2±8.7  (31.0-84.0) | 57.3±9.0 | 57.9±8.3 | 62.5±8.4 | <0.001 | Kruskal-Wallis |
| cesd10 | Continuous Variable | 12.1±6.5  (0.0-29.0) | 9.4±5.9 | 11.9±6.3 | 13.8±6.6 | <0.001 | Kruskal-Wallis |
| srh | Categorical Variable | 61 (10.8%) | 3 (3.1%) | 29 (9.8%) | 29 (16.9%) | <0.001 | Chi-square |
| eyesight_distance | Categorical Variable | 189 (33.5%) | 20 (20.6%) | 89 (30.1%) | 80 (46.5%) | <0.001 | Chi-square |
| Edu | Categorical Variable | 357 (63.2%) | 46 (47.4%) | 188 (63.5%) | 123 (71.5%) | 0.0044 | Chi-square |
| Iadl | Categorical Variable | 398 (70.4%) | 85 (87.6%) | 215 (72.6%) | 98 (57.0%) | <0.001 | Chi-square |
| adlab_c | Categorical Variable | 385 (68.1%) | 81 (83.5%) | 216 (73.0%) | 88 (51.2%) | <0.001 | Chi-square |
| glass | Categorical Variable | 506 (89.6%) | 90 (92.8%) | 269 (90.9%) | 147 (85.5%) | 0.2246 | Chi-square |
| gender | Categorical Variable | 392 (69.4%) | 61 (62.9%) | 205 (69.3%) | 126 (73.3%) | 0.2077 | Chi-square |
| hibpe | Categorical Variable | 409 (72.4%) | 84 (86.6%) | 212 (71.6%) | 113 (65.7%) | 0.001 | Chi-square |
| diabe | Categorical Variable | 530 (93.8%) | 95 (97.9%) | 280 (94.6%) | 155 (90.1%) | 0.0274 | Chi-square |
| hearte | Categorical Variable | 460 (81.4%) | 89 (91.8%) | 246 (83.1%) | 125 (72.7%) | <0.001 | Chi-square |
| psyche | Categorical Variable | 557 (98.6%) | 97 (100.0%) | 293 (99.0%) | 167 (97.1%) | 0.1067 | Chi-square |
| arthre | Categorical Variable | 209 (37.0%) | 62 (63.9%) | 103 (34.8%) | 44 (25.6%) | <0.001 | Chi-square |
| asthmae | Categorical Variable | 529 (93.6%) | 96 (99.0%) | 281 (94.9%) | 152 (88.4%) | 0.0012 | Chi-square |
| drinkev | Categorical Variable | 374 (66.2%) | 57 (58.8%) | 193 (65.2%) | 124 (72.1%) | 0.0743 | Chi-square |
| smokev | Categorical Variable | 423 (74.9%) | 70 (72.2%) | 218 (73.6%) | 135 (78.5%) | 0.4048 | Chi-square |
| disability | Categorical Variable | 428 (75.8%) | 86 (88.7%) | 224 (75.7%) | 118 (68.6%) | 0.0011 | Chi-square |
| hip | Categorical Variable | 553 (97.9%) | 95 (97.9%) | 289 (97.6%) | 169 (98.3%) | 0.9031 | Chi-square |
| ins | Categorical Variable | 18 (3.2%) | 7 (7.2%) | 10 (3.4%) | 1 (0.6%) | 0.0115 | Chi-square |
| da042s1 | Categorical Variable | 390 (69.0%) | 77 (79.4%) | 199 (67.2%) | 114 (66.3%) | 0.0518 | Chi-square |
| da042s12 | Categorical Variable | 386 (68.3%) | 81 (83.5%) | 207 (69.9%) | 98 (57.0%) | <0.001 | Chi-square |
| ea001s7 | Categorical Variable | 560 (99.1%) | 96 (99.0%) | 294 (99.3%) | 170 (98.8%) | 0.851 | Chi-square |
| ea001s8 | Categorical Variable | 560 (99.1%) | 96 (99.0%) | 292 (98.6%) | 172 (100.0%) | 0.3177 | Chi-square |
| ea001s11 | Categorical Variable | 556 (98.4%) | 95 (97.9%) | 290 (98.0%) | 171 (99.4%) | 0.4461 | Chi-square |

**Supplementary Table 3** Baseline demographic and clinical characteristics of participants

| **Characteristics** | | | | | | | | **Robust** | | | **Pre-frailty** | | | | | | | | **Frailty** | | | | | |  |  |  |  |
| --- | --- | --- | --- | --- | --- | --- | --- | --- | --- | --- | --- | --- | --- | --- | --- | --- | --- | --- | --- | --- | --- | --- | --- | --- | --- | --- | --- | --- |
| **Age, mean (SD)** | | | | | | | |  | | |  | | | | | | | |  | | | | | |  |  |  |  |
| 3 years-frailty | | | | | | | | 57.13(8.39) | | | 58.78(8.32) | | | | | | | | 62.11(9.29) | | | | | |  |  |  |  |
| 6 years-frailty | | | | | | | | 57.28(9.01) | | | 58.86(8.27) | | | | | | | | 62.48(8.43) | | | | | |  |  |  |  |
| **Vision, mean (SD)** | | | | | | | |  | | |  | | | | | | | |  | | | | | |  |  |  |  |
| 3 years-frailty | | | | | | | | 2.25(0.88) | | | 1.96(0.92) | | | | | | | | 1.75(0.79) | | | | | |  |  |  |  |
| 6 years-frailty | | | | | | | | 2.19(0.83) | | | 2.06(0.94) | | | | | | | | 1.70(0.79) | | | | | |  |  |  |  |
| **Mental health, mean (SD)** | | | | | | | |  | | |  | | | | | | | |  | | | | | |  |  |  |  |
| 3 years-frailty | | | | | | | | 8.52(5.43) | | | 12.43(6.16) | | | | | | | | 14.54(6.72) | | | | | |  |  |  |  |
| 6 years-frailty | | | | | | | | 9.37(5.92) | | | 11.92(6.27) | | | | | | | | 13.80(6.57) | | | | | |  |  |  |  |
| **ADL, mean (SD)** | | | | | | | |  | | |  | | | | | | | |  | | | | | |  |  |  |  |
| 3 years-frailty | | | | | | | | 0.24(0.68) | | | 0.50(0.97) | | | | | | | | 1.35(1.66) | | | | | |  |  |  |  |
| 6 years-frailty | | | | | | | | 0.21(0.52) | | | 0.47(0.96) | | | | | | | | 1.16(1.55) | | | | | |  |  |  |  |
| **IADL, mean (SD)** | | | | | | | |  | | |  | | | | | | | |  | | | | | |  |  |  |  |
| 3 years-frailty | | | | | | | | 0.20(0.62) | | | 0.50(0.98) | | | | | | | | 1.33(1.60) | | | | | |  |  |  |  |
| 6 years-frailty | | | | | | | | 0.19(0.55) | | | 0.49(0.97) | | | | | | | | 1.09(1.51) | | | | | |  |  |  |  |
| **Self rated health, mean (SD)** | | | | | | | |  | | |  | | | | | | | |  | | | | | |  |  |  |  |
| 3 years-frailty | | | | | | | | 2.80(0.77) | | | 2.49(0.79) | | | | | | | | 2.14(0.80) | | | | | |  |  |  |  |
| 6 years-frailty | | | | | | | | 2.89(0.75) | | | 2.49(0.79) | | | | | | | | 2.22(0.79) | | | | | |  |  |  |  |
| **Gender, n (%)** | | | | | | | |  | | |  | | | | | | | |  | | | | | |  |  |  |  |
| 3 years-frailty | | | | | | | |  | | |  | | | | | | | |  | | | | | |  |  |  |  |
| Female | | | | | | | | 47(8.32%) | | | 96(16.99%) | | | | | | | | 30(5.31%) | | | | | |  |  |  |  |
| Male | | | | | | | | 75(13.27%) | | | 221(39.12%) | | | | | | | | 96(16.99%) | | | | | |  |  |  |  |
| 6 years-frailty | | | | | | | |  | | |  | | | | | | | |  | | | | | |  |  |  |  |
| Female | | | | | | | | 36(6.37%) | | | 91(16.11%) | | | | | | | | 46(8.14%) | | | | | |  |  |  |  |
| Male | | | | | | | | 61(10.80%) | | | 205(36.28%) | | | | | | | | 126(22.30%) | | | | | |  |  |  |  |
| **Education, n (%)** | | | | | |  | | | | | | | |  | |  | | | | | | | | | | | |  |
| 3 years-frailty | | | | | |  | | | | | | | |  | |  | | | | | | | | | | | |  |
| Yes | | | | | | 56(9.91%) | | | | | | | | 202(35.75%) | | 99(17.52%) | | | | | | | | | | | |  |
| No | | | | | | NA | | | | | | | | NA | | NA | | | | | | | | | | | |  |
| 6 years-frailty | | | | | |  | | | | | | | |  | |  | | | | | | | | | | | |  |
| Yes | | | | | | 279(11.57%) | | | | | | | | 1087(45.07%) | | 729(30.22%) | | | | | | | | | | | |  |
| No | | | | | | 24(1.00%) | | | | | | | | 130(5.39%) | | 163(6.76%) | | | | | | | | | | | |  |
| **Hypertension, n (%)** | | | | | | | |  | | |  | | | | | | | |  | | | | | |  |  |  |  |
| 3 years-frailty | | | | | | | |  | | |  | | | | | | | |  | | | | | |  |  |  |  |
| Yes | | | | | | | | 15(2.65%) | | | 91(16.11%) | | | | | | | | 50(8.85%) | | | | | |  |  |  |  |
| No | | | | | | | | 107(18.94%) | | | 226(40.00%) | | | | | | | | 76(13.45%) | | | | | |  |  |  |  |
| 6 years-frailty | | | | | | | |  | | |  | | | | | | | |  | | | | | |  |  |  |  |
| Yes | | | | | | | | 13(2.30%) | | | 84(14.87%) | | | | | | | | 59(10.44%) | | | | | |  |  |  |  |
| No | | | | | | | | 84(14.87%) | | | 212(37.52%) | | | | | | | | 113(20.00%) | | | | | |  |  |  |  |
| **Hearte, n (%)** | | | | | | | |  | | |  | | | | | | | |  | | | | | |  |  |  |  |
| 3 years-frailty | | | | | | | |  | | |  | | | | | | | |  | | | | | |  |  |  |  |
| Yes | | | | | | | | 7(1.24%) | | | 65(11.50%) | | | | | | | | 33(5.84%) | | | | | |  |  |  |  |
| No | | | | | | | | 115(20.35%) | | | 252(44.60%) | | | | | | | | 93(16.46%) | | | | | |  |  |  |  |
| 6 years-frailty | | | | | | | |  | | |  | | | | | | | |  | | | | | |  |  |  |  |
| Yes | | | | | | | | 8(1.42%) | | | 50(8.85%) | | | | | | | | 47(8.32%) | | | | | |  |  |  |  |
| No | | | | | | | | 89(15.75%) | | | 246(43.54%) | | | | | | | | 125(22.12%) | | | | | |  |  |  |  |
| **Diabetes, n (%)** | | | | | | | |  | | |  | | | | | | | |  | | | | | |  |  |  |  |
| 3 years-frailty | | | | | | | |  | | |  | | | | | | | |  | | | | | |  |  |  |  |
| Yes | | | | | | | | NA | | | 21(3.72%) | | | | | | | | 14(2.48%) | | | | | |  |  |  |  |
| No | | | | | | | | 122(21.59%) | | | 296(52.39%) | | | | | | | | 112(19.82%) | | | | | |  |  |  |  |
| 6 years-frailty | | | | | | | |  | | |  | | | | | | | |  | | | | | |  |  |  |  |
| Yes | | | | | | | | 2(0.35%) | | | 16(2.83%) | | | | | | | | 17(3.01%) | | | | | |  |  |  |  |
| No | | | | | | | | 95(16.81%) | | | 280(49.56%) | | | | | | | | 155(27.43%) | | | | | |  |  |  |  |
| **Mental Illness, n (%)** | | | | | | | |  | | |  | | | | | | | |  | | | | | |  |  |  |  |
| 3 years-frailty | | | | | | | |  | | |  | | | | | | | |  | | | | | |  |  |  |  |
| Yes | | | | | | | | NA | | | 4(0.71%) | | | | | | | | 4(0.71%) | | | | | |  |  |  |  |
| No | | | | | | | | 122(21.59%) | | | 313(55.40%) | | | | | | | | 122(21.59%) | | | | | |  |  |  |  |
| 6 years-frailty | | | | | | | |  | | |  | | | | | | | |  | | | | | |  |  |  |  |
| Yes | | | | | | | | NA | | | 3(0.53%) | | | | | | | | 5(0.88%) | | | | | |  |  |  |  |
| No | | | | | | | | 97(17.17%) | | | 293(51.86%) | | | | | | | | 167(29.56%) | | | | | |  |  |  |  |
| **Arthritis, n (%)** | | | | | | | |  | | |  | | | | | | | |  | | | | | |  |  |  |  |
| 3 years-frailty | | | | | | | |  | | |  | | | | | | | |  | | | | | |  |  |  |  |
| Yes | | | | | | | | 50(8.85%) | | | 207(36.64%) | | | | | | | | 99(17.52%) | | | | | |  |  |  |  |
| No | | | | | | | | 72(12.74%) | | | 110(19.47%) | | | | | | | | 27(4.78%) | | | | | |  |  |  |  |
| 6 years-frailty | | | | | | | |  | | |  | | | | | | | |  | | | | | |  |  |  |  |
| Yes | | | | | | | | 35(6.19%) | | | 193(34.16%) | | | | | | | | 128(22.65%) | | | | | |  |  |  |  |
| No | | | | | | | | 62(10.97%) | | | 103(18.23%) | | | | | | | | 44(7.79%) | | | | | |  |  |  |  |
| **Hip Fracture, n (%)** | | | | | | | | |  |  | | | | | | | | | | | |  | |  |  |  |  |  |
| 3 years-frailty | | | | | | | | |  |  | | | | | | | | | | | |  | |  |  |  |  |  |
| Yes | | | | | | | | | 2(0.35%) | 5(0.88%) | | | | | | | | | | | | 5(0.88%) | |  |  |  |  |  |
| No | | | | | | | | | 120(21.24%) | 312(55.22%) | | | | | | | | | | | | 121(21.42%) | |  |  |  |  |  |
| 6 years-frailty | | | | | | | | |  |  | | | | | | | | | | | |  | |  |  |  |  |  |
| Yes | | | | | | | | | 2(0.35%) | 7(1.24%) | | | | | | | | | | | | 3(0.53%) | |  |  |  |  |  |
| No | | | | | | | | | 95(16.81%) | 289(51.15%) | | | | | | | | | | | | 169(29.91%) | |  |  |  |  |  |
| **Knee Pain, n (%)** | | | | | | | |  | | |  | | | | | | | |  | | | | | |  |  |  |  |
| 3 years-frailty | | | | | | | |  | | |  | | | | | | | |  | | | | | |  |  |  |  |
| Yes | | | | | | | | 21(3.72%) | | | 97(17.17%) | | | | | | | | 61(10.80%) | | | | | |  |  |  |  |
| No | | | | | | | | 101(17.88%) | | | 220(38.94%) | | | | | | | | 65(11.50%) | | | | | |  |  |  |  |
| 6 years-frailty | | | | | | | |  | | |  | | | | | | | |  | | | | | |  |  |  |  |
| Yes | | | | | | | | 16(2.83%) | | | 89(15.75%) | | | | | | | | 74(13.10%) | | | | | |  |  |  |  |
| No | | | | | | | | 81(14.34%) | | | 207(36.64%) | | | | | | | | 98(17.35%) | | | | | |  |  |  |  |
| **Asthmae, n (%)** | | | | | | | |  | | |  | | | | | | | |  | | | | | |  |  |  |  |
| 3 years-frailty | | | | | | | |  | | |  | | | | | | | |  | | | | | |  |  |  |  |
| Yes | | | | | | | | 4(0.71%) | | | 19(3.36%) | | | | | | | | 13(2.30%) | | | | | |  |  |  |  |
| No | | | | | | | | 118(20.88%) | | | 298(52.74%) | | | | | | | | 113(20.00%) | | | | | |  |  |  |  |
| 6 years-frailty | | | | | | | |  | | |  | | | | | | | |  | | | | | |  |  |  |  |
| Yes | | | | | | | | 1(0.18%) | | | 15(2.65%) | | | | | | | | 20(3.54%) | | | | | |  |  |  |  |
| No | | | | | | | | 96(16.99%) | | | 281(49.73%) | | | | | | | | 152(26.90%) | | | | | |  |  |  |  |
| **Drink, n (%)** | | | | | | | |  | | |  | | | | | | | |  | | | | | |  |  |  |  |
| 3 years-frailty | | | | | | | |  | | |  | | | | | | | |  | | | | | |  |  |  |  |
| Yes | | | | | | | | 46(8.14%) | | | 106(18.76%) | | | | | | | | 39(6.90%) | | | | | |  |  |  |  |
| No | | | | | | | | 76(13.45%) | | | 211(37.35%) | | | | | | | | 87(15.40%) | | | | | |  |  |  |  |
| 6 years-frailty | | | | | | | |  | | |  | | | | | | | |  | | | | | |  |  |  |  |
| Yes | | | | | | | | 40(7.08%) | | | 103(18.23%) | | | | | | | | 48(8.50%) | | | | | |  |  |  |  |
| No | | | | | | | | 57(10.09%) | | | 193(34.16%) | | | | | | | | 124(21.95%) | | | | | |  |  |  |  |
| **Smoke, n (%)** | | | | | |  | | | | |  | | | | | |  | | | | | | | |  |  |  |  |
| 3 years-frailty | | | | | |  | | | | |  | | | | | |  | | | | | | | |  |  |  |  |
| Yes | | | | | | 35(6.19%) | | | | | 87(15.40%) | | | | | | 37(6.55%) | | | | | | | |  |  |  |  |
| No | | | | | | 70(12.39%) | | | | | 218(38.58%) | | | | | | 135(23.89%) | | | | | | | |  |  |  |  |
| 6 years-frailty | | | | | |  | | | | |  | | | | | |  | | | | | | | |  |  |  |  |
| Yes | | | | 95(31.35%) | | | | | | | | | 311(25.55%) | | | | | | 181(20.29%) | | | | | |  |  |  |  |
| No | | | | 208(68.65%) | | | | | | | | | 906(74.45%) | | | | | | 711(79.71%) | | | | | |  |  |  |  |
| **Medical Insurance Ownership, n (%)** | | | | |  | | | | | | | | |  | | |  | | | | | | | | | |  |  |
| 3 years-frailty | | | | |  | | | | | | | | |  | | |  | | | | | | | | | |  |  |
| Yes | | | | | 115(20.35%) | | | | | | | | | 306(54.16%) | | | 126(22.30%) | | | | | | | | | |  |  |
| No | | | | | 7(1.24%) | | | | | | | | | 11(1.95%) | | | NA | | | | | | | | | |  |  |
| 6 years-frailty | | | | |  | | | | | | | | |  | | |  | | | | | | | | | |  |  |
| Yes | | | | | 90(15.93%) | | | | | | | | | 286(50.62%) | | | 171(30.27%) | | | | | | | | | |  |  |
| No | | | | | 7(1.24%) | | | | | | | | | 10(1.77%) | | | 1(0.18%) | | | | | | | | | |  |  |
| **Urban Employee Medical Insurance, n (%)** | | | |  | | | | | | | | | |  | | | | |  | | | | | | | | |  |
| 3 years-frailty | | | |  | | | | | | | | | |  | | | | |  | | | | | | | | |  |
| Yes | | | | NA | | | | | | | | | | 1(0.18%) | | | | | 4(0.71%) | | | | | | | | |  |
| No | | | | 122(21.59%) | | | | | | | | | | 316(55.93%) | | | | | 122(21.59%) | | | | | | | | |  |
| 6 years-frailty | | | |  | | | | | | | | | |  | | | | |  | | | | | | | | |  |
| Yes | | | | 1(0.18%) | | | | | | | | | | 2(0.35%) | | | | | 2(0.35%) | | | | | | | | |  |
| No | | | | 96(16.99%) | | | | | | | | | | 294(47.31%) | | | | | 170(30.09%) | | | | | | | | |  |
| **Private Medical Insurance, n (%)** | | | |  | | | | | | | | | |  | | | | |  | | | | | | | | |  |
| 3 years-frailty | | | |  | | | | | | | | | |  | | | | |  | | | | | | | | |  |
| Yes | | | | 3(0.53%) | | | | | | | | | | 2(0.35%) | | | | | NA | | | | | | | | |  |
| No | | | | 119(21.06%) | | | | | | | | | | 315(55.75%) | | | | | 126(22.30%) | | | | | | | | |  |
| 6 years-frailty | | | |  | | | | | | | | | |  | | | | |  | | | | | | | | |  |
| Yes | | | | 1(0.18%) | | | | | | | | | | 4(0.71%) | | | | | NA | | | | | | | | |  |
| No | | | | 96(16.99%) | | | | | | | | | | 292(51.68%) | | | | | 172(30.44%) | | | | | | | | |  |
| **Other Medical Insurance, n (%)** | | |  | | | | | | | | | | | |  | | | |  | | | | | | | | | |
| 3 years-frailty | | |  | | | | | | | | | | | |  | | | |  | | | | | | | | | |
| Yes | | | 2(0.35%) | | | | | | | | | | | | 4(0.71%) | | | | 3(0.53%) | | | | | | | | | |
| No | | | 120(21.24%) | | | | | | | | | | | | 313(55.40%) | | | | 123(21.77%) | | | | | | | | | |
| 6 years-frailty | | |  | | | | | | | | | | | |  | | | |  | | | | | | | | | |
| Yes | | | 2(0.35%) | | | | | | | | | | | | 6(1.06%) | | | | 1(0.18%) | | | | | | | | | |
| No | | | 95(16.81%) | | | | | | | | | | | | 290(51.33%) | | | | 171(30.27%) | | | | | | | | | |
| **Disability, n (%)** | | |  | | | | | | | | | |  | | | | | | | |  | | | |  |  |  |  |
| 3 years-frailty | | |  | | | | | | | | | |  | | | | | | | |  | | | |  |  |  |  |
| Yes | | | 9(1.59%) | | | | | | | | | | 86(15.22%) | | | | | | | | 42(7.43%) | | | |  |  |  |  |
| No | | | 113(20.00%) | | | | | | | | | | 231(40.88%) | | | | | | | | 84(14.87%) | | | |  |  |  |  |
| 6 years-frailty | |  | | | | | | | | | | |  | | | | | | | |  | | | |  |  |  |  |
| Yes | | 11(1.95%) | | | | | | | | | | | 72(12.74%) | | | | | | | | 54(9.56%) | | | |  |  |  |  |
| No | | 86(15.22%) | | | | | | | | | | | 224(39.65%) | | | | | | | | 118(20.88%) | | | |  |  |  |  |
| **Glass, n (%)** | |  | | | | | | | | | | |  | | | | | | | |  | | | |  |  |  |  |
| 3 years-frailty | |  | | | | | | | | | | |  | | | | | | | |  | | | |  |  |  |  |
| Yes | | 8(1.42%) | | | | | | | | | | | 34(6.02%) | | | | | | | | | | 14(2.48%) | |  |  |  |  |
| No | | 114(20.18%) | | | | | | | | | | | 281(49.73%) | | | | | | | | | | 111(19.65%) | |  |  |  |  |
| 6 years-frailty | |  | | | | | | | | | | |  | | | | | | | |  | | | |  |  |  |  |
| Yes | | 7(1.24%) | | | | | | | | | | | 26(4.60%) | | | | | | | | 23(4.07%) | | | |  |  |  |  |
| No | | 90(15.93%) | | | | | | | | | | | 269(47.61%) | | | | | | | | 147(26.02%) | | | |  |  |  |  |

**Supplementary Table 4** Performance Metrics of Single Models for 3-Year Frailty Index Prediction

| **Model** | **Dataset** | **Accuracy (95%*CI*)** | **Precision** | **F1-score** | **Recall** |
| --- | --- | --- | --- | --- | --- |
| Tab Transformer | Train set | 0.9801  (0.9626-0.9895) | 0.9808 | 0.9799 | 0.9801 |
|  | Test set | 0.5487 (0.4568-0.6373) | 0.5324 | 0.5232 | 0.5487 |
| CNN | Train set | 0.9358 (0.9094-0.9550) | 0.9403 | 0.9347 | 0.9358 |
|  | Test set | 0.5841 (0.4919-0.6707) | 0.5833 | 0.5816 | 0.5841 |
| DNN | Train set | 0.9912 (0.9775-0.9966) | 0.9913 | 0.9912 | 0.9912 |
|  | Test set | 0.5487 (0.4568-0.6373) | 0.5422 | 0.5428 | 0.5487 |
| RF | Train set | 1.0000 (0.9916-1.0000) | 1.0000 | 1.0000 | 1.0000 |
|  | Test set | 0.6018 (0.5096-0.6872) | 0.6151 | 0.5748 | 0.6018 |
| XGBoost | Train set | 1.0000 (0.9916-1.0000) | 1.0000 | 1.0000 | 1.0000 |
|  | Test set | 0.5752 (0.4831-0.6624) | 0.5757 | 0.5616 | 0.5752 |

**Supplementary Table 5** Performance Metrics of Single Models for 6-Year Frailty Index Prediction

| **Model** | **Dataset** | **Accuracy**  **(95%*CI*)** | **Precision** | **F1-score** | **Recall** |
| --- | --- | --- | --- | --- | --- |
| Tab Transformer | Train set | 0.9823  (0.9655-0.9910) | 0.9825 | 0.9823 | 0.9823 |
|  | Test set | 0.4779  (0.3880-0.5692) | 0.4733 | 0.4745 | 0.4779 |
| CNN | Train set | 0.9336  (0.9068-0.9531) | 0.938 | 0.9306 | 0.9336 |
|  | Test set | 0.5929  (0.5007-0.6790) | 0.6173 | 0.596 | 0.5929 |
| DNN | Train set | 0.9956  (0.9840-0.9988) | 0.9956 | 0.9956 | 0.9956 |
|  | Test set | 0.4956  (0.4051-0.5864) | 0.5037 | 0.4989 | 0.4956 |
| RF | Train set | 1.0000  (0.9916-1.0000) | 1.0000 | 1.0000 | 1.0000 |
|  | Test set | 0.5575  (0.4656-0.6457) | 0.5506 | 0.5484 | 0.5575 |
| XGBoost | Train set | 1.0000  (0.9916-1.0000) | 1.0000 | 1.0000 | 1.0000 |
|  | Test set | 0.5487  (0.4568-0.6373) | 0.5517 | 0.5494 | 0.5487 |

**
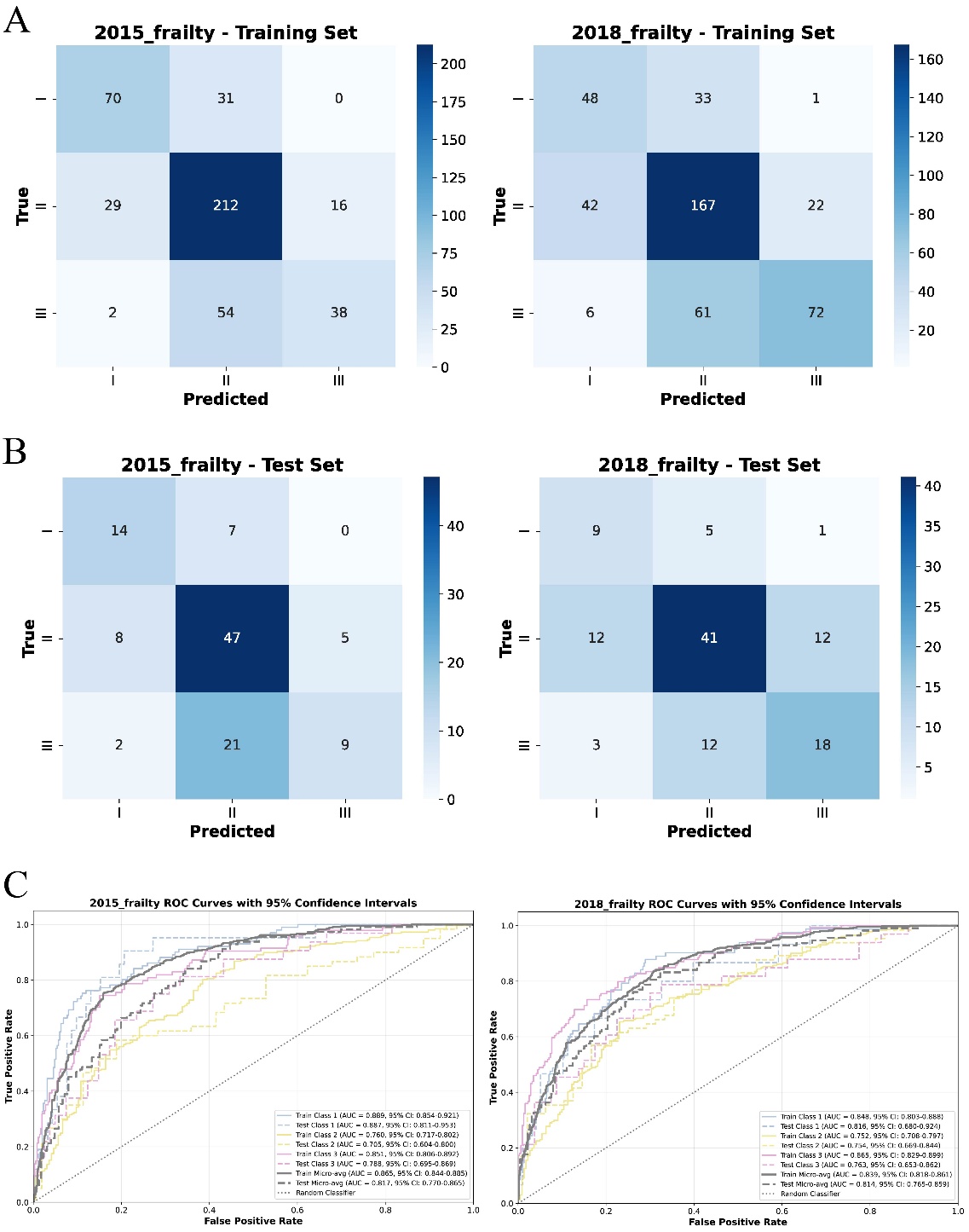
Supplementary Figure 1** **Confusion Matrices and ROC Curves of the MMoE model using CNN’s experts for Frailty Prediction at 3 and 6 Years.** (A) Confusion matrices for the training set at 3-year and 6-year intervals, showing predictions for Robust, Pre-frail, and Frail categories. (B) Confusion matrices for the test set at the corresponding intervals. (C) ROC curves for each prediction interval, with ROC values indicating the model’s discriminatory ability between classes (AUC, area under the curve; CI, Confidence interval; Micro-avg, Micro-averaging; ROC, Receiver Operating Characteristic Curve).


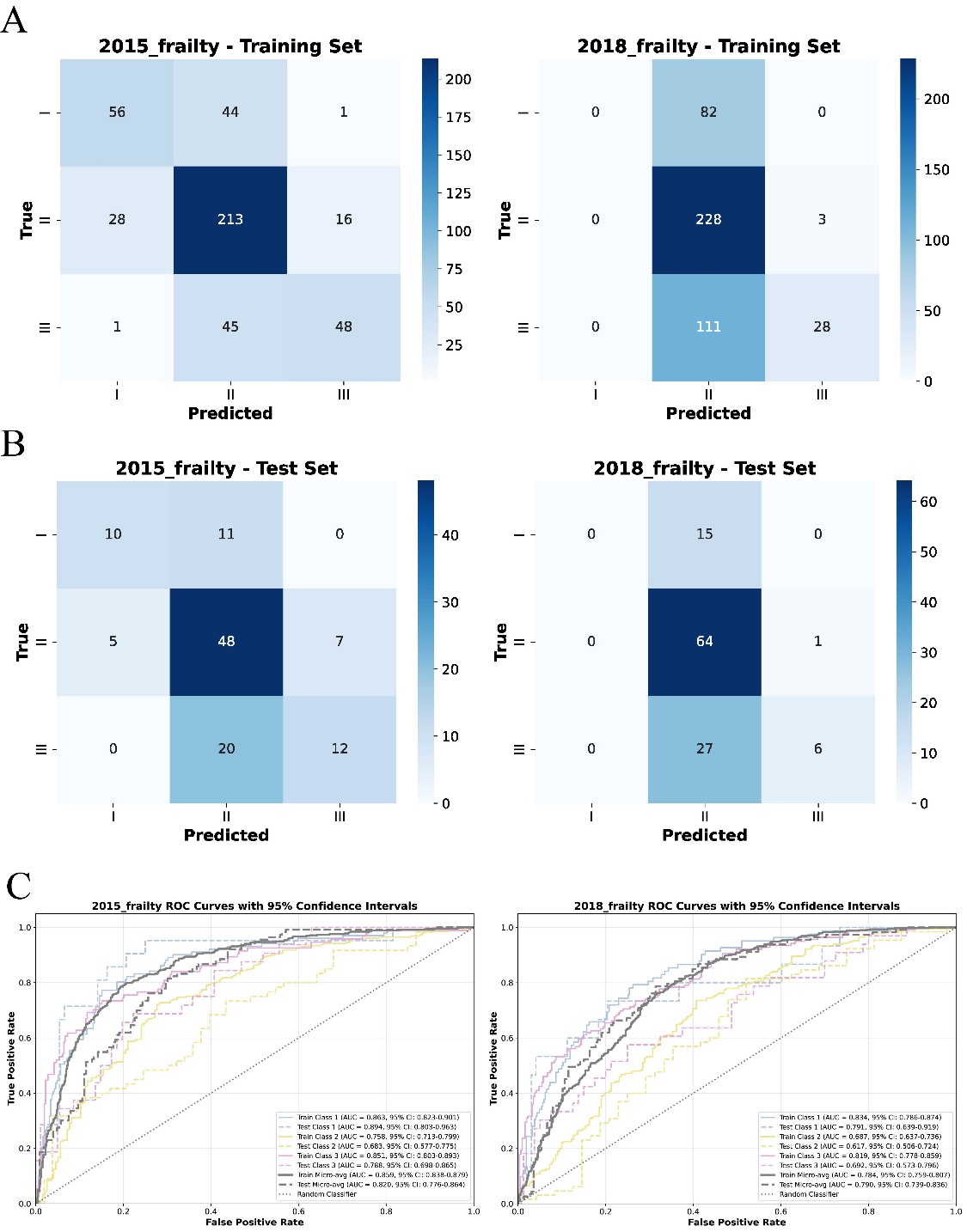
**Supplementary Figure 2** **Confusion Matrices and ROC Curves of the MMoE model using DNN’s experts for Frailty Prediction at 3 and 6 Years.** (A) Confusion matrices for the training set at 3-year and 6-year intervals, showing predictions for Robust, Pre-frail, and Frail categories. (B) Confusion matrices for the test set at the corresponding intervals. (C) ROC curves for each prediction interval, with ROC values indicating the model’s discriminatory ability between classes (AUC, area under the curve; CI, Confidence interval; Micro-avg, Micro-averaging; ROC, Receiver Operating Characteristic Curve).

**
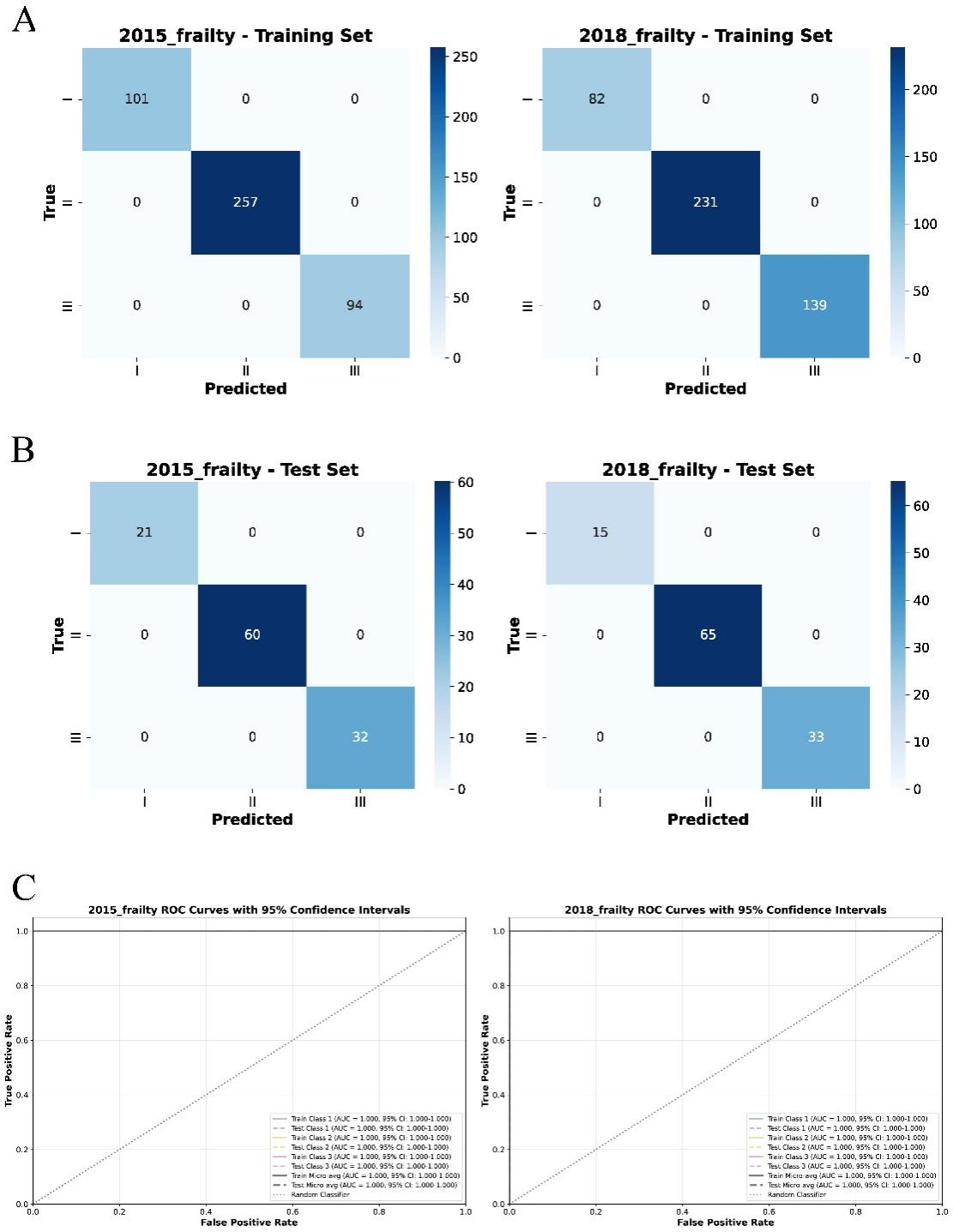
Supplementary Figure 3** **Confusion Matrices and ROC Curves of the MMoE model using RF’s experts for Frailty Prediction at 3 and 6 Years.** (A) Confusion matrices for the training set at 3-year and 6-year intervals, showing predictions for Robust, Pre-frail, and Frail categories. (B) Confusion matrices for the test set at the corresponding intervals. (C) ROC curves for each prediction interval, with ROC values indicating the model’s discriminatory ability between classes (AUC, area under the curve; CI, Confidence interval; Micro-avg, Micro-averaging; ROC, Receiver Operating Characteristic Curve).

**
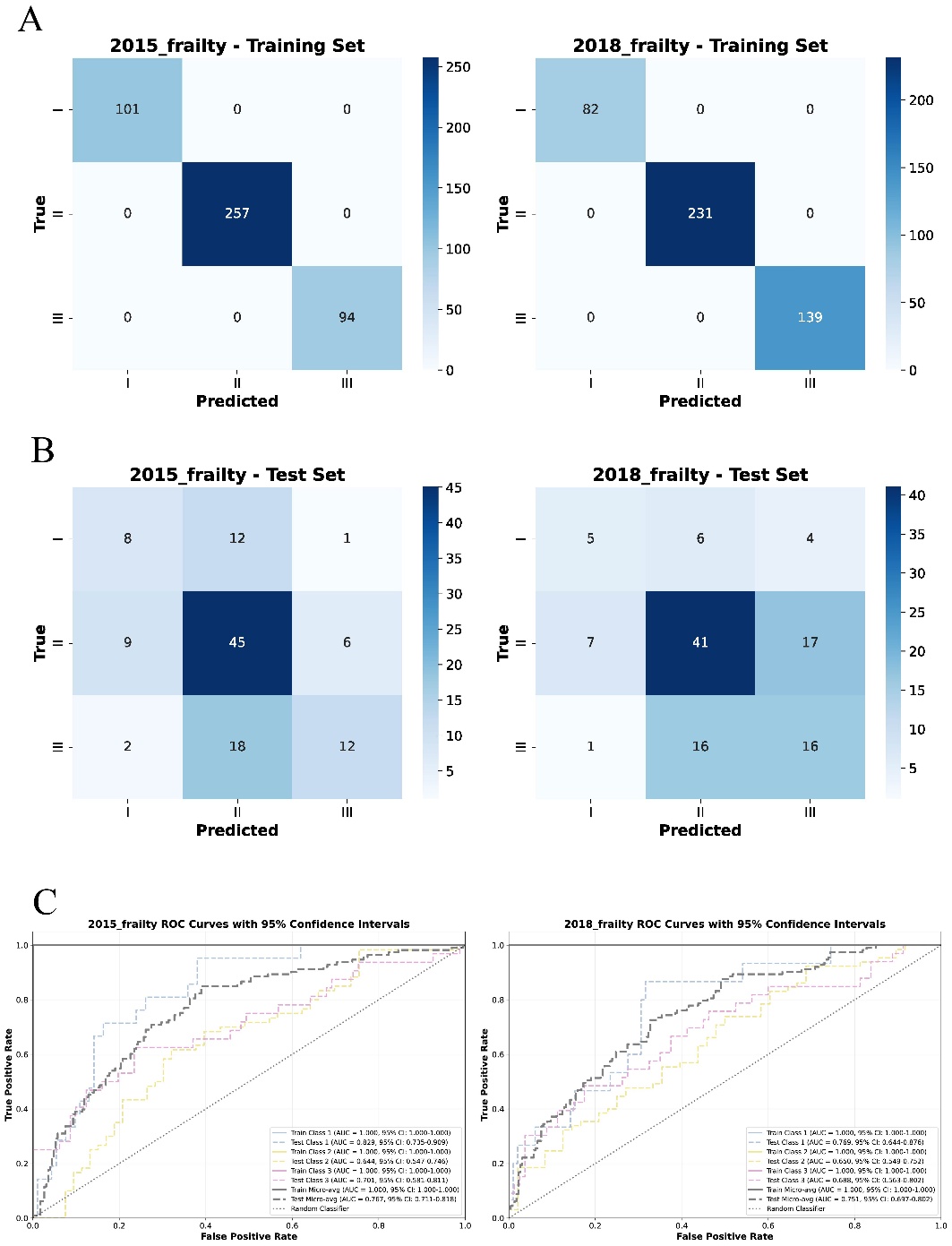
**

**Supplementary Figure 4** **Confusion Matrices and ROC Curves of the MMoE model using XGBoost’s experts for Frailty Prediction at 3 and 6 Years.** (A) Confusion matrices for the training set at 3-year and 6-year intervals, showing predictions for Robust, Pre-frail, and Frail categories. (B) Confusion matrices for the test set at the corresponding intervals. (C) ROC curves for each prediction interval, with ROC values indicating the model’s discriminatory ability between classes (AUC, area under the curve; CI, Confidence interval; Micro-avg, Micro-averaging; ROC, Receiver Operating Characteristic Curve).


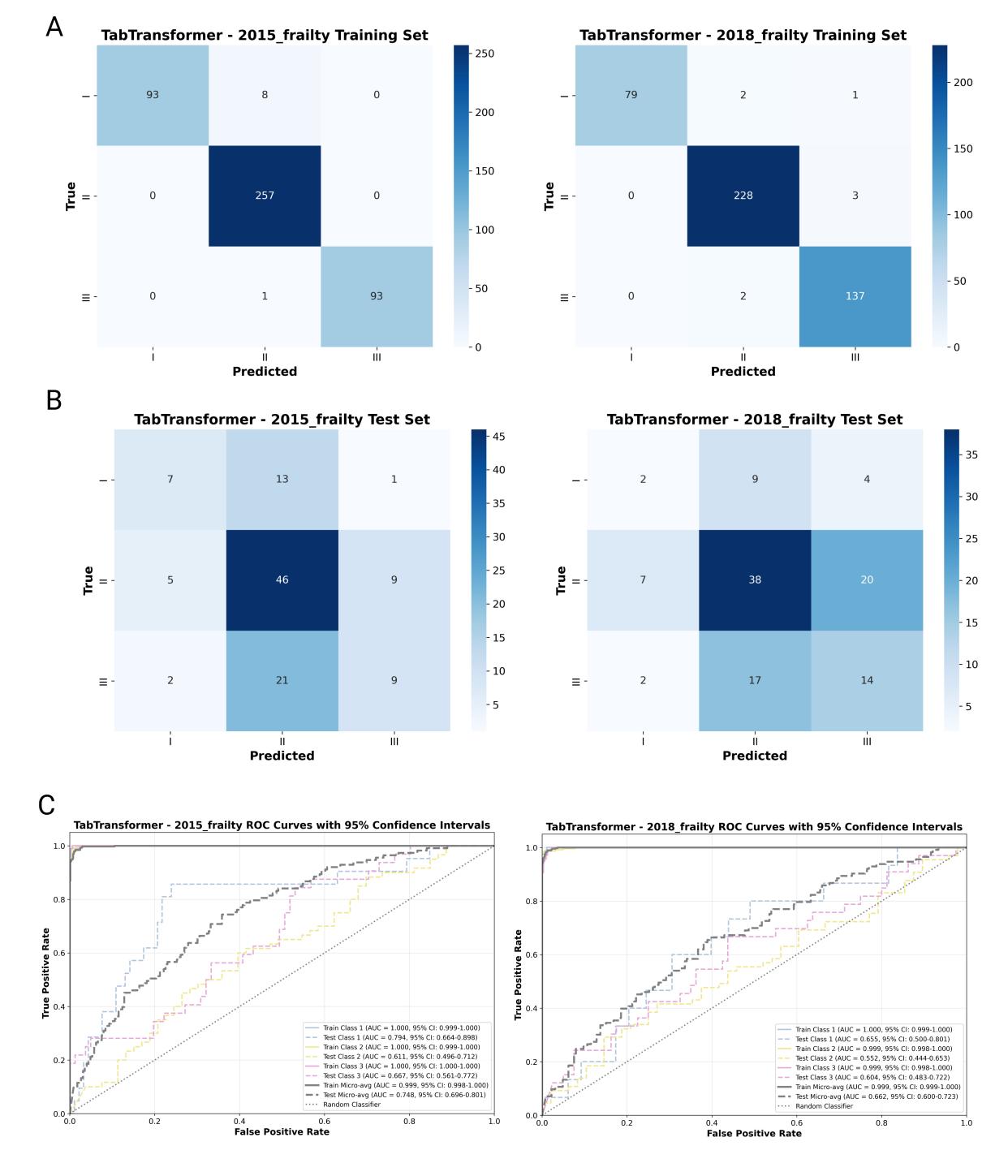
**Supplementary Figure 5 Confusion Matrices and ROC Curves of the Tab Transformer Single Model for Frailty Prediction at 3 and 6 Years.** (A) Confusion matrices for the training set at 3-year and 6-year intervals, showing predictions for Robust, Pre-frail, and Frail categories. (B) Confusion matrices for the test set at the corresponding intervals. (C) ROC curves for each prediction interval, with ROC values indicating the model’s discriminatory ability between classes (AUC, area under the curve; CI, Confidence interval; Micro-avg, Micro-averaging; ROC, Receiver Operating Characteristic Curve).


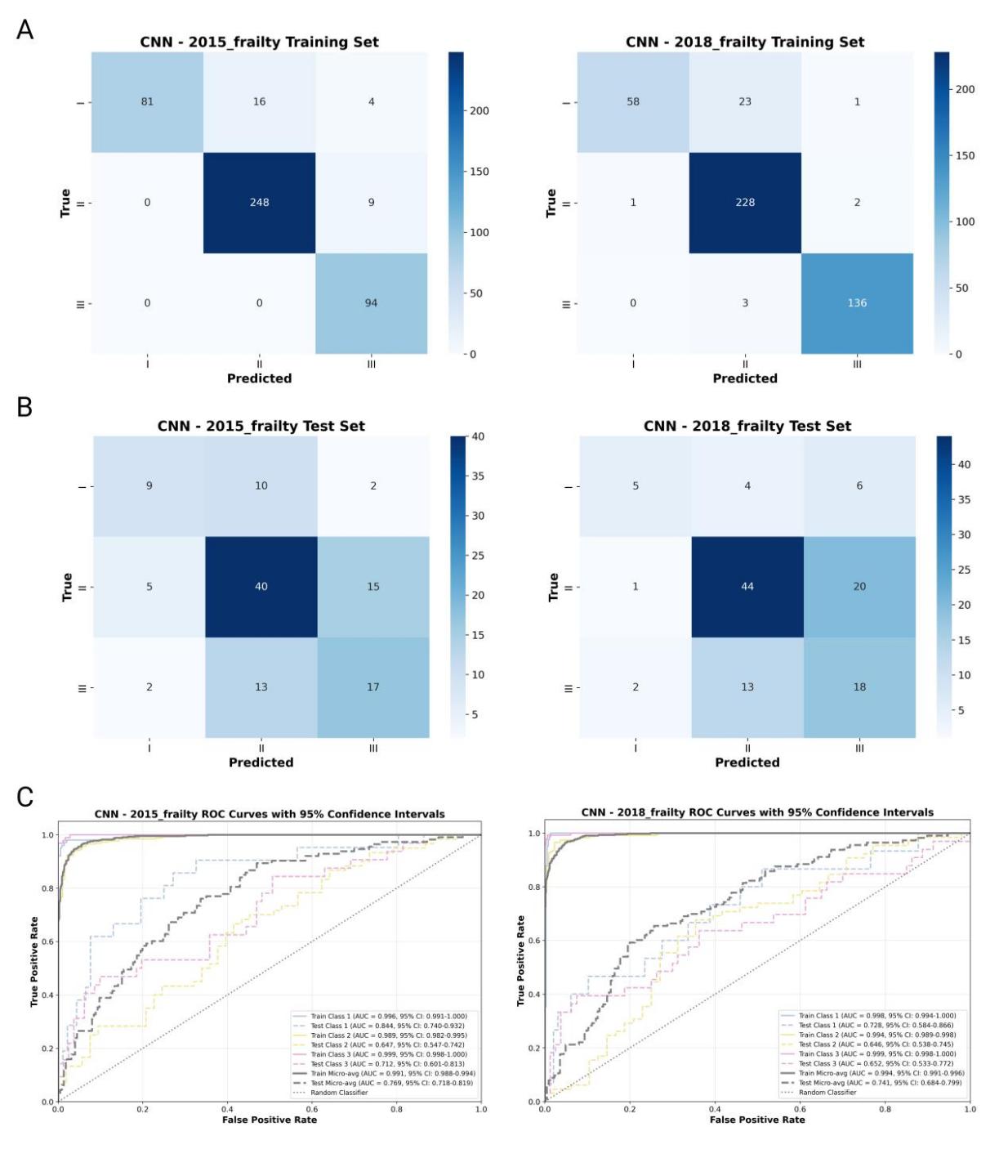
**Supplementary Figure 6 Confusion Matrices and ROC Curves of the CNN Single Model for Frailty Prediction at 3 and 6 Years**. (A) Confusion matrices for the training set at 3-year and 6-year intervals, showing predictions for Robust, Pre-frail, and Frail categories. (B) Confusion matrices for the test set at the corresponding intervals. (C) ROC curves for each prediction interval, with ROC values indicating the model’s discriminatory ability between classes (AUC, area under the curve; CI, Confidence interval; Micro-avg, Micro-averaging; ROC, Receiver Operating Characteristic Curve).


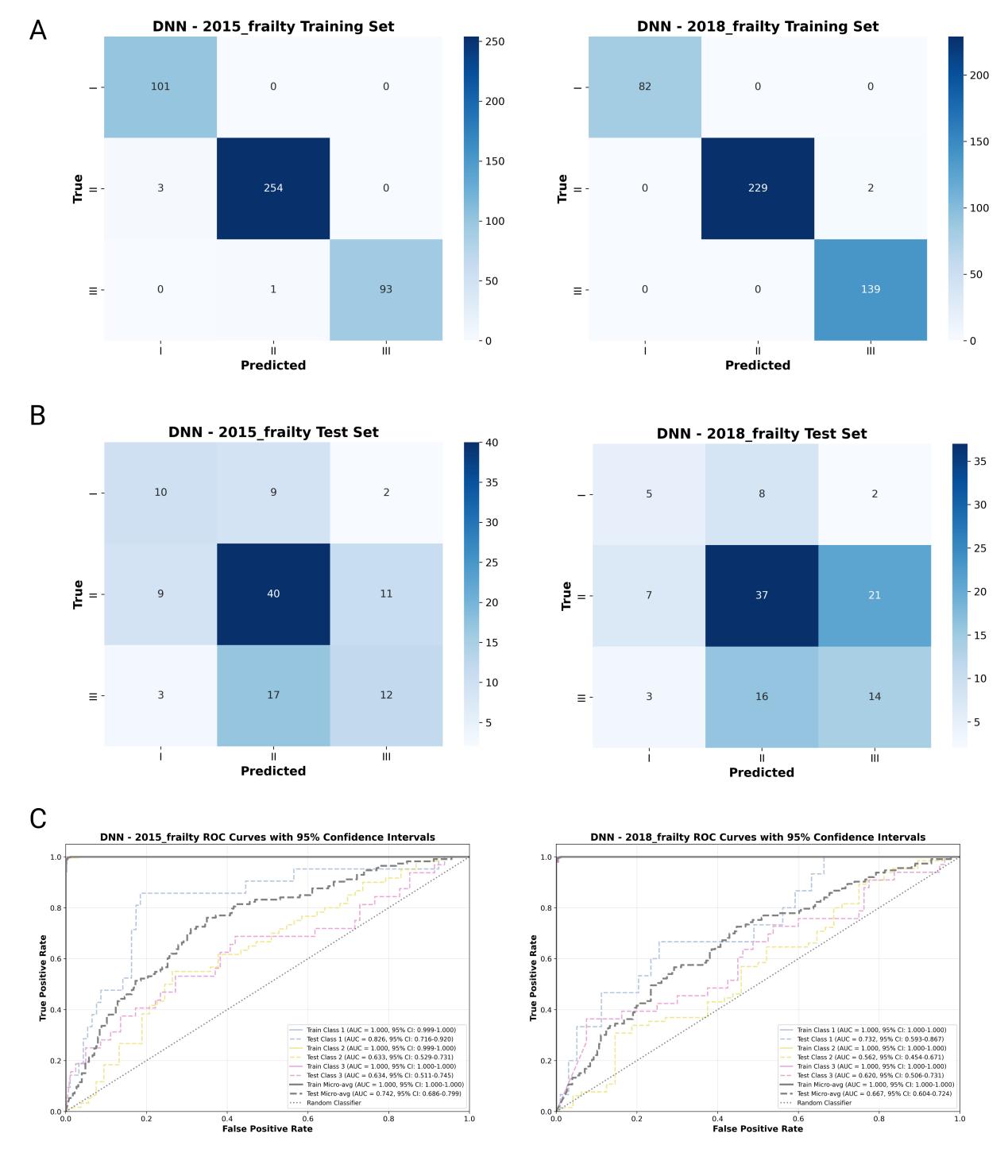
**Supplementary Figure 7 Confusion Matrices and ROC Curves of the DNN Single Model for Frailty Prediction at 3 and 6 Years.** (A) Confusion matrices for the training set at 3-year and 6-year intervals, showing predictions for Robust, Pre-frail, and Frail categories. (B) Confusion matrices for the test set at the corresponding intervals. (C) ROC curves for each prediction interval, with ROC values indicating the model’s discriminatory ability between classes (AUC, area under the curve; CI, Confidence interval; Micro-avg, Micro-averaging; ROC, Receiver Operating Characteristic Curve).


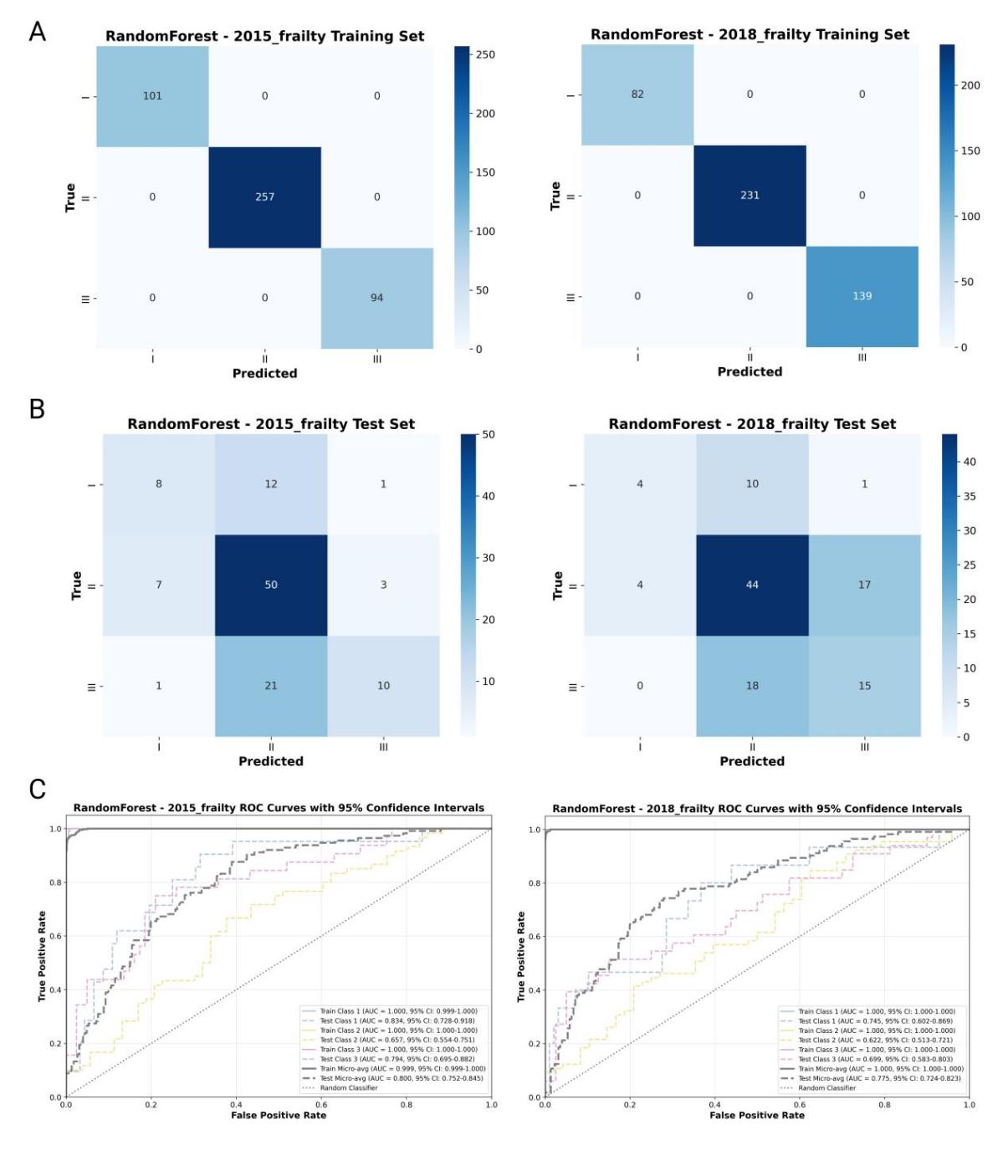
**Supplementary Figure 8 Confusion Matrices and ROC Curves of the Random Forest Single Model for Frailty Prediction at 3 and 6 Years.** (A) Confusion matrices for the training set at 3-year and 6-year intervals, showing predictions for Robust, Pre-frail, and Frail categories. (B) Confusion matrices for the test set at the corresponding intervals. (C) ROC curves for each prediction interval, with ROC values indicating the model’s discriminatory ability between classes (AUC, area under the curve; CI, Confidence interval; Micro-avg, Micro-averaging; ROC, Receiver Operating Characteristic Curve).


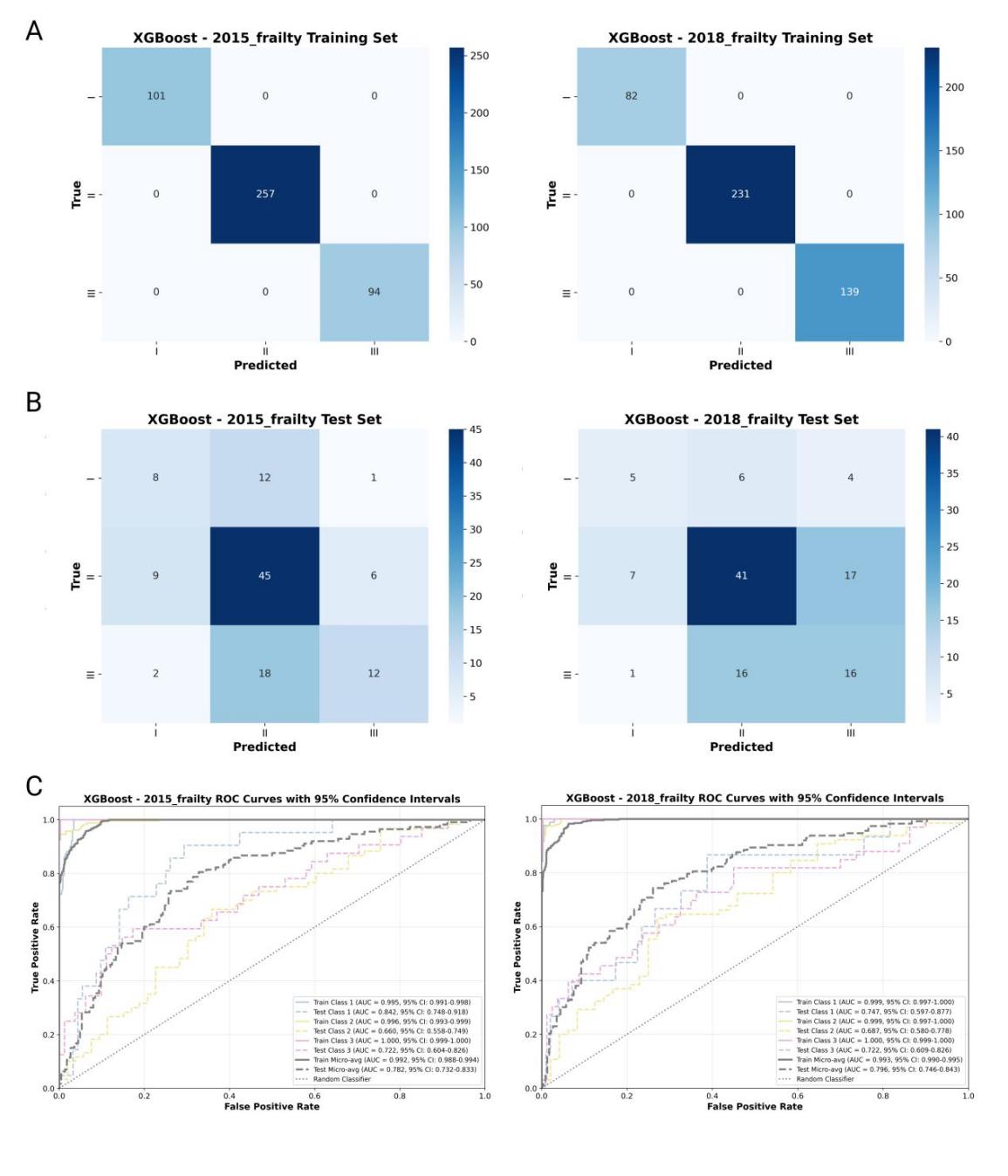
**Supplementary Figure 9 Confusion Matrices and ROC Curves of the XGBoost Single Model for Frailty Prediction at 3 and 6 Years.** (A) Confusion matrices for the training set at 3-year and 6-year intervals, showing predictions for Robust, Pre-frail, and Frail categories. (B) Confusion matrices for the test set at the corresponding intervals. (C) ROC curves for each prediction interval, with ROC values indicating the model’s discriminatory ability between classes (AUC, area under the curve; CI, Confidence interval; Micro-avg, Micro-averaging; ROC, Receiver Operating Characteristic Curve).

**
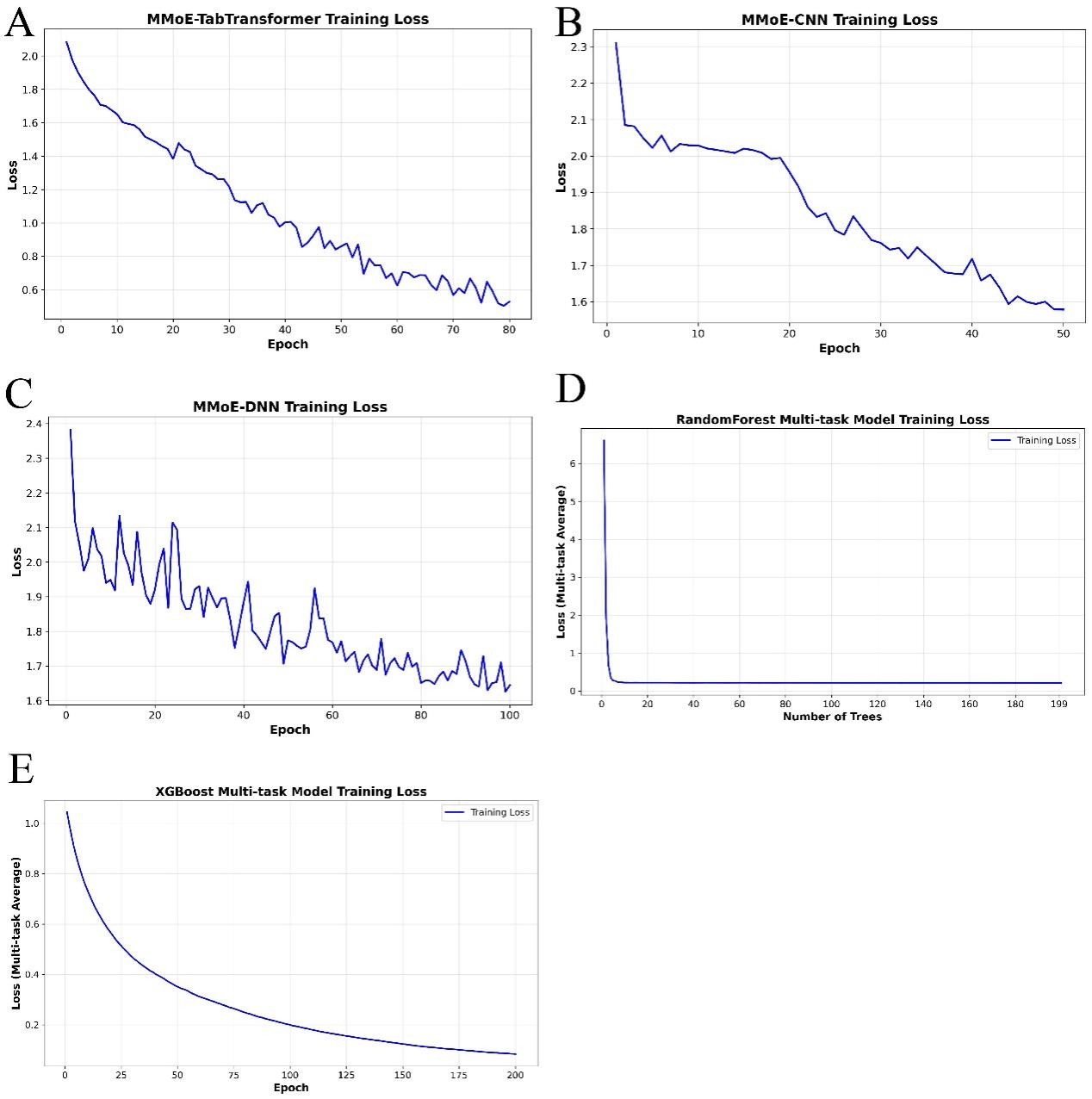
**

**Supplementary Figure 10 Training Loss of the MMoE Models with Five Different Experts for Frailty Prediction.** (A) Training Loss of the MMoE model using Tab Transformer ’s experts. (B) Training Loss of the MMoE model using CNN ’s Experts. (C) Training Loss of the MMoE model using DNN ’s Experts. (D) Training Loss of the MMoE model using RF ’s Experts. (E) Training Loss of the MMoE model using XGBoost ’s Experts.

**
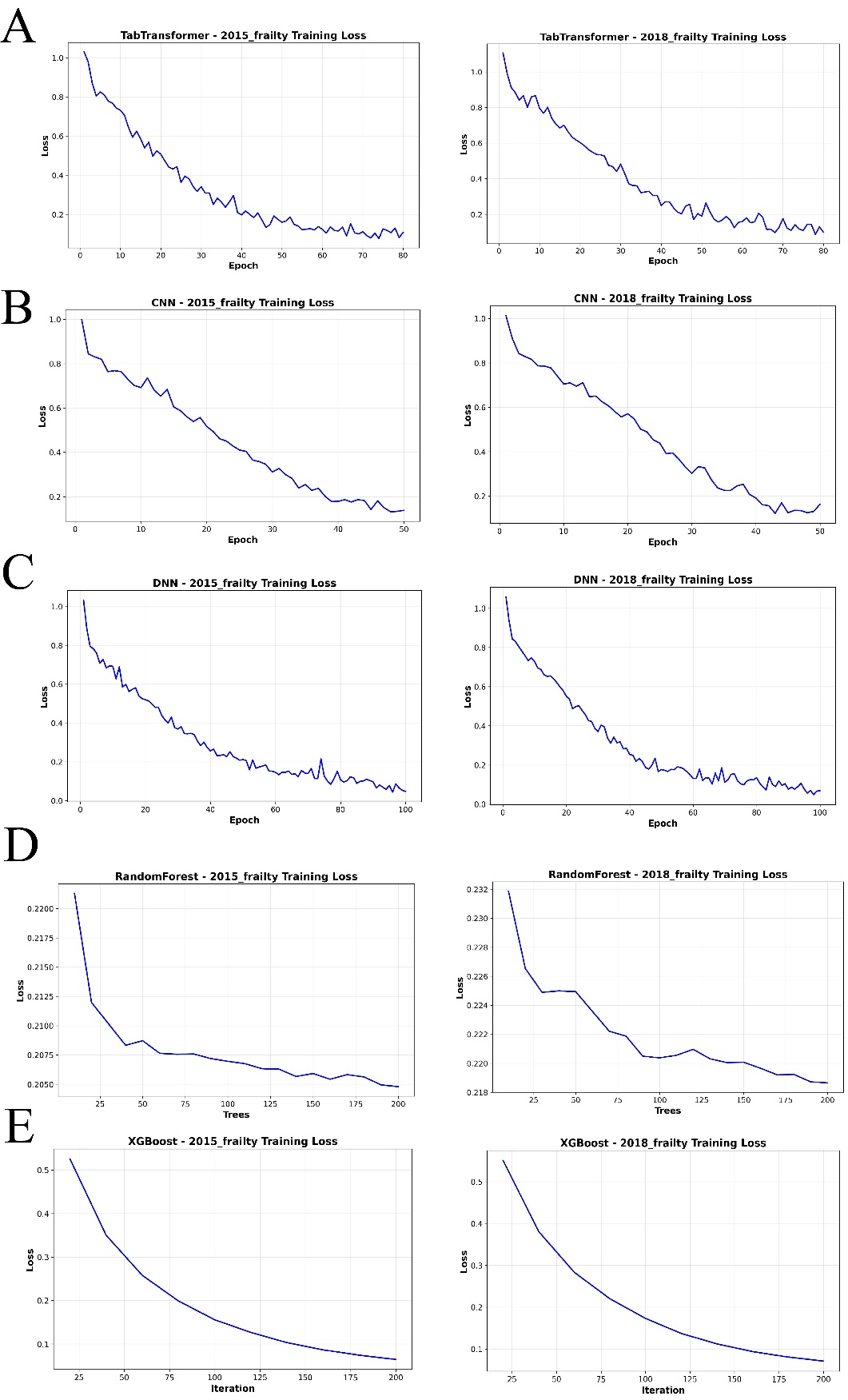
Supplementary Figure 11 Training Loss of the Five Different Single Models for Frailty Prediction at 3 and 6 Years.** (A) Training Loss of the Tab Transformer Single Model**.** (B) Training Loss of the CNN Single Model. (C) Training Loss of the DNN Single Model. (D) Training Loss of the RF Single Model. (E) Training Loss of the XGBoost Single Model.

**
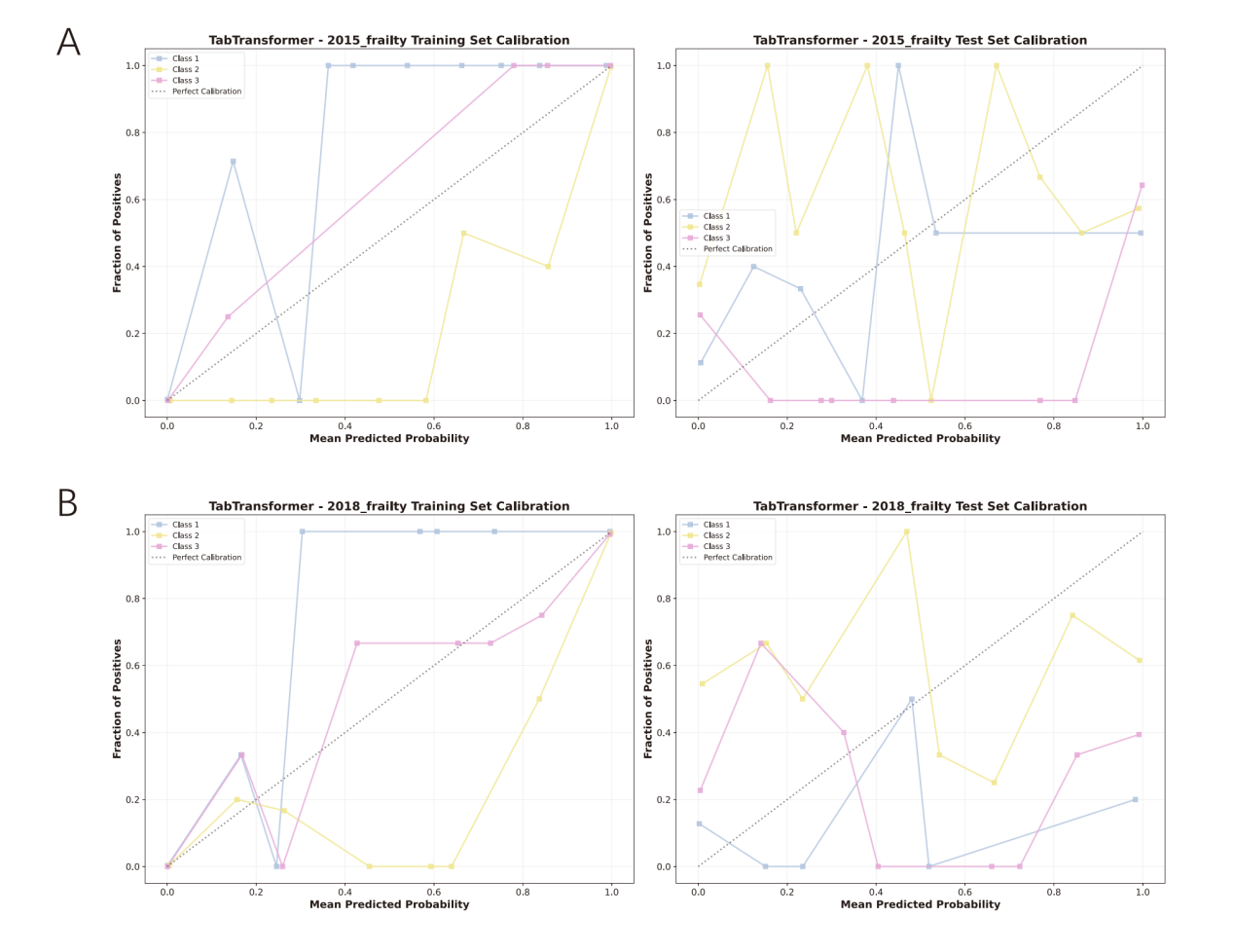
**

**Supplementary Figure 12 Calibration of the TabTransformer Single Model for Frailty Prediction at 3 and 6 Years**

**
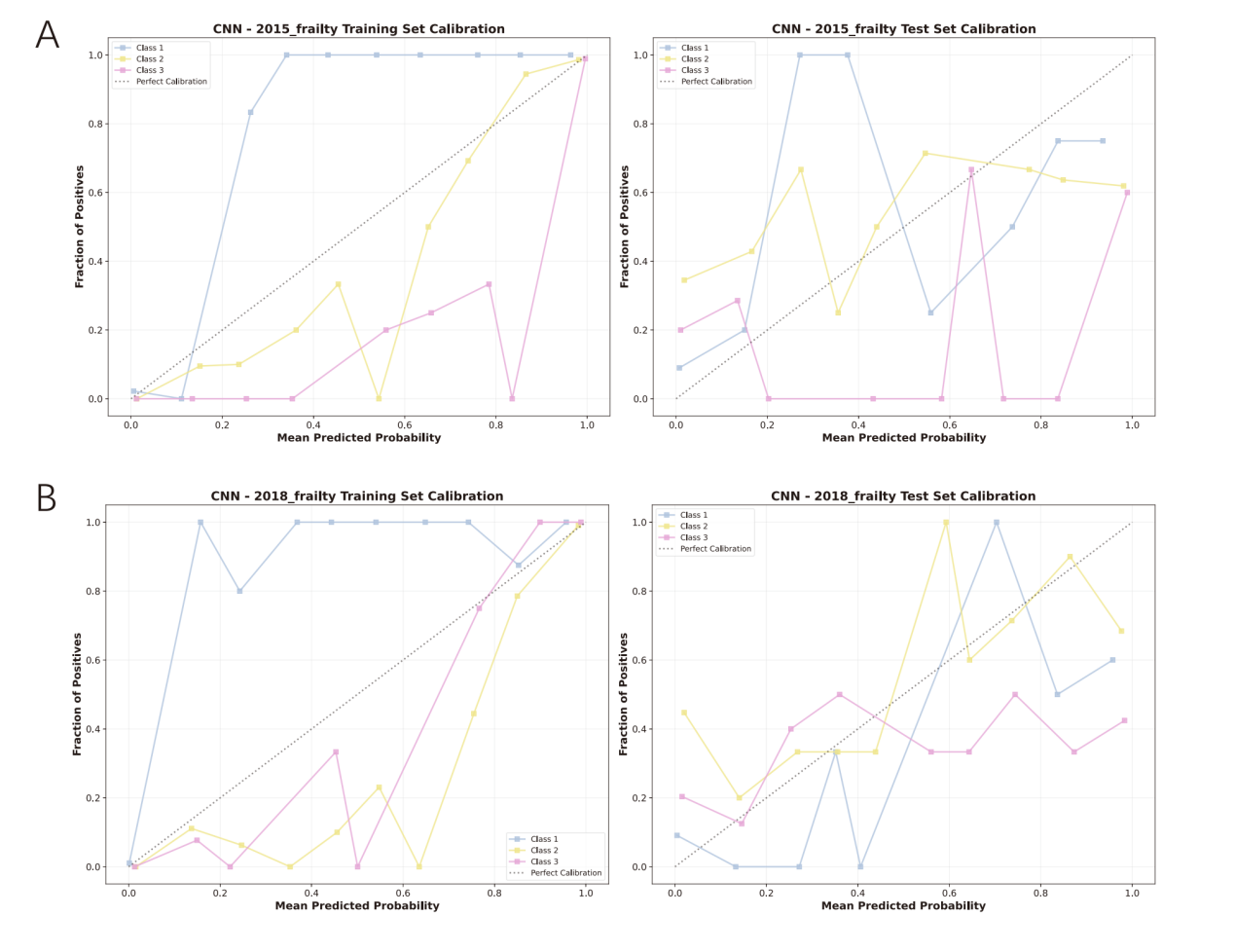
Supplementary Figure 13 Calibration of the CNN Single Model for Frailty Prediction at 3 and 6 Years**

**
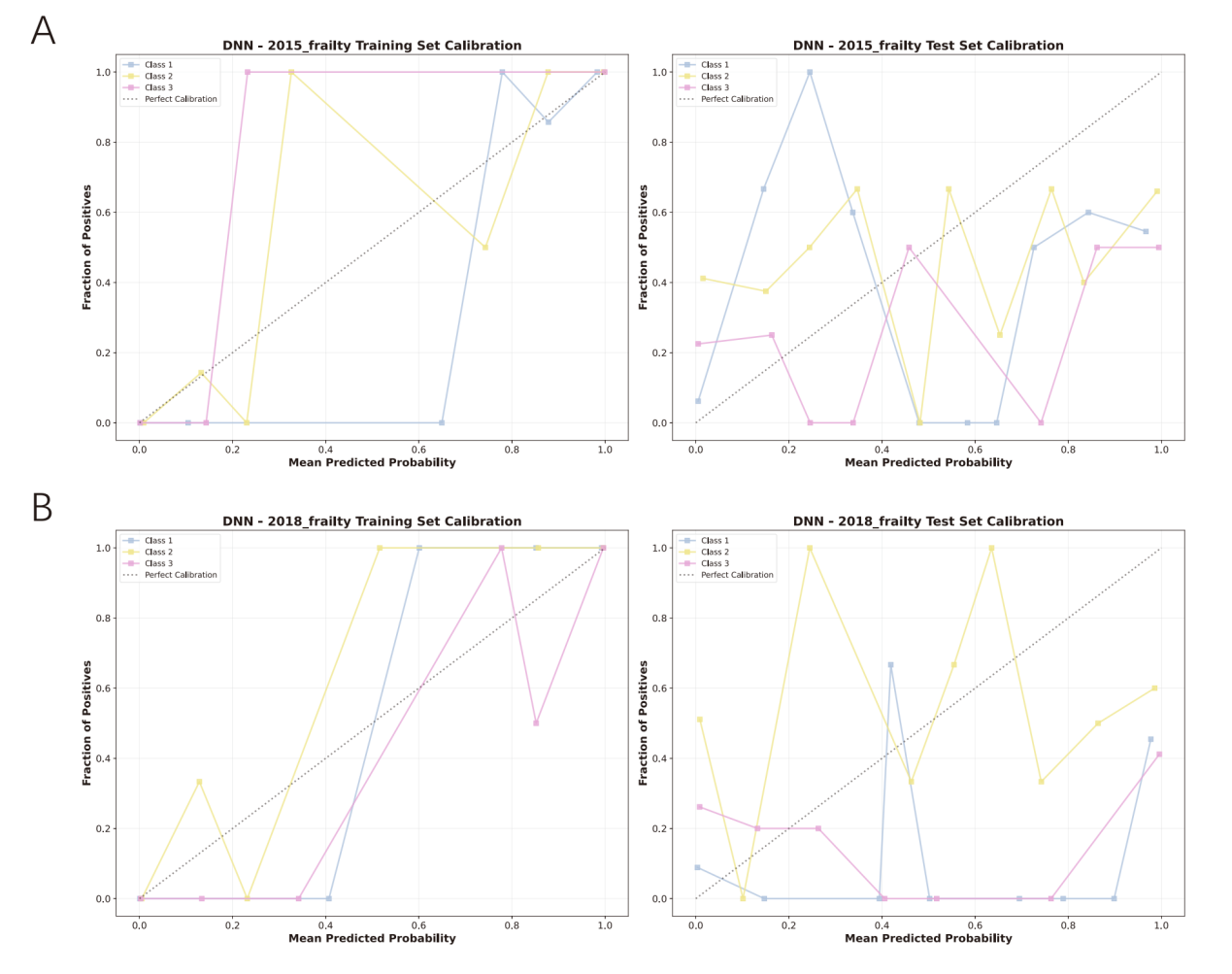
**

**Supplementary Figure 14 Calibration of the DNN Single Model for Frailty Prediction at 3 and 6 Years**

**
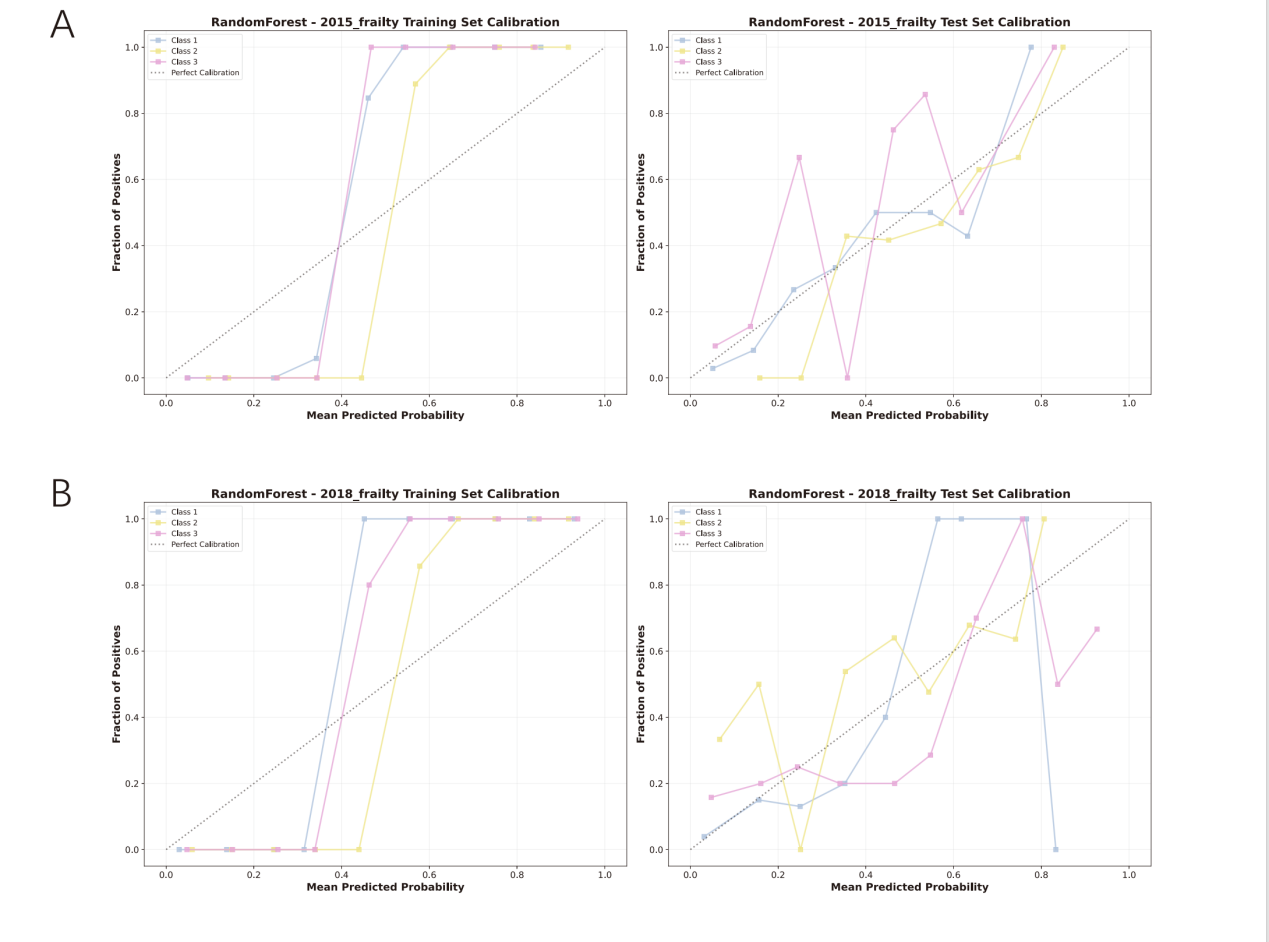
**

**Supplementary Figure 15 Calibration of the Random Forest Single Model for Frailty Prediction at 3 and 6 Years**

**
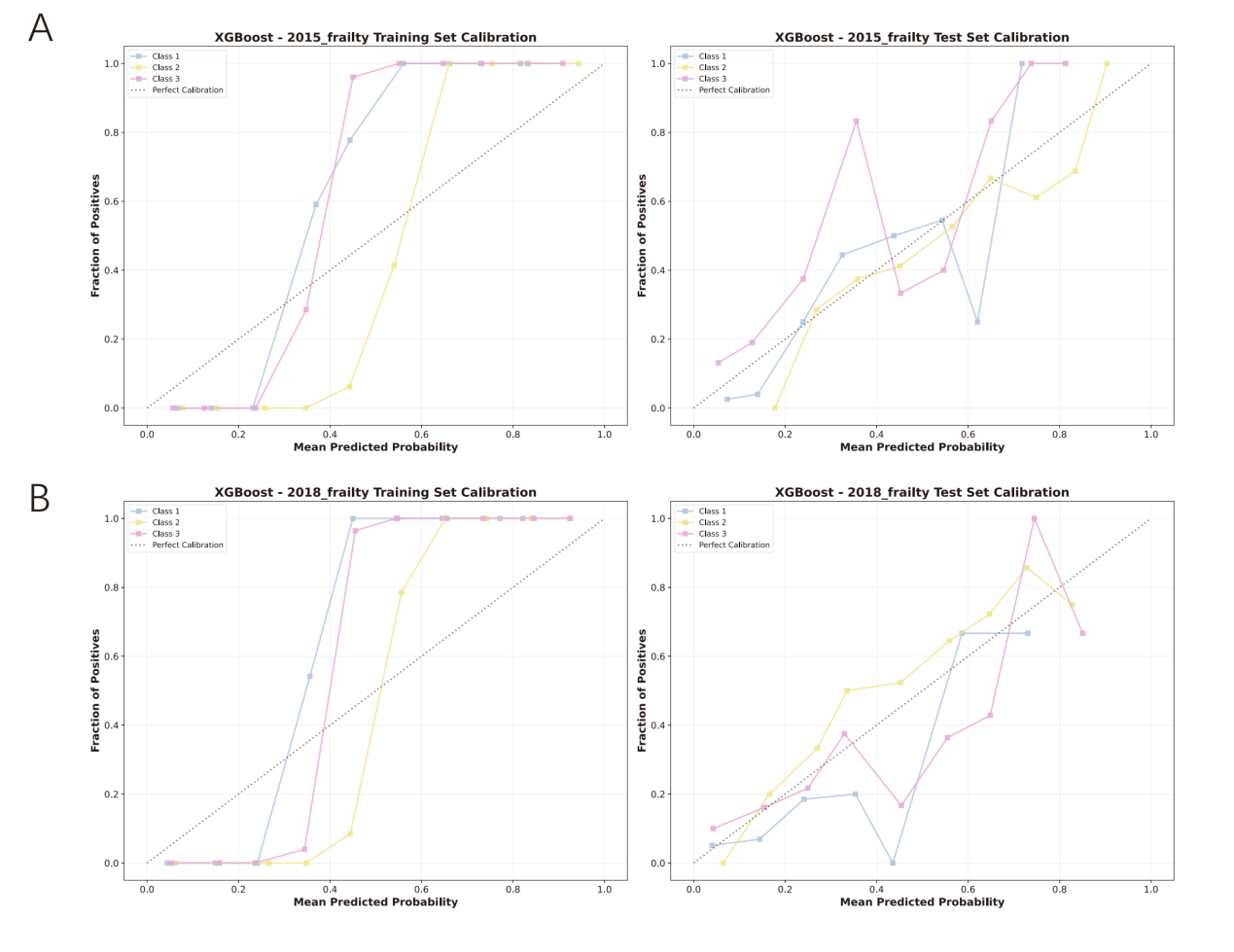
**

**Supplementary Figure 16 Calibration of the XGBoost Single Model for Frailty Prediction at 3 and 6 Years**

**
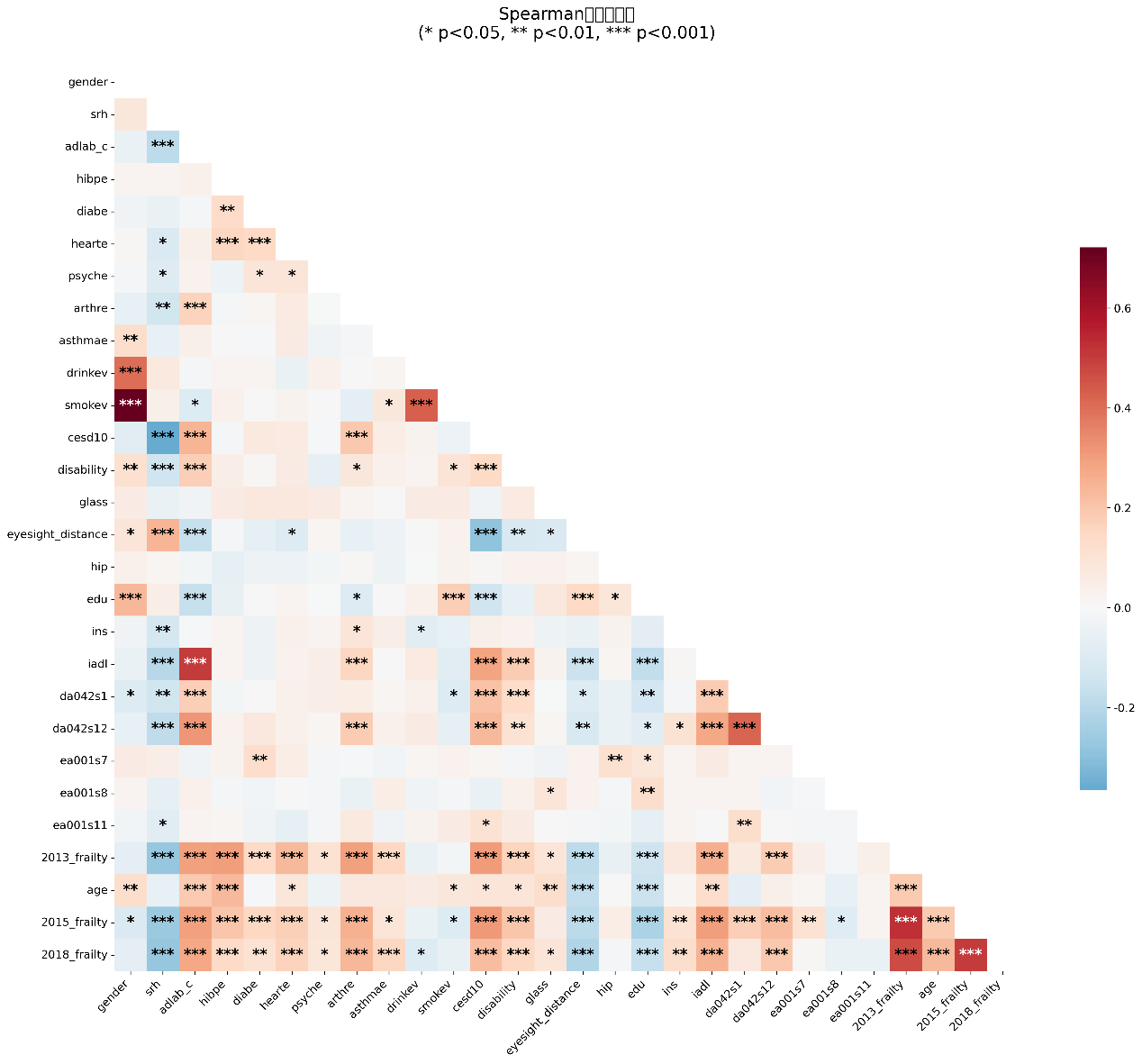
**

**Supplementary Figure 17 Spearman Correlation Heatmap**

**
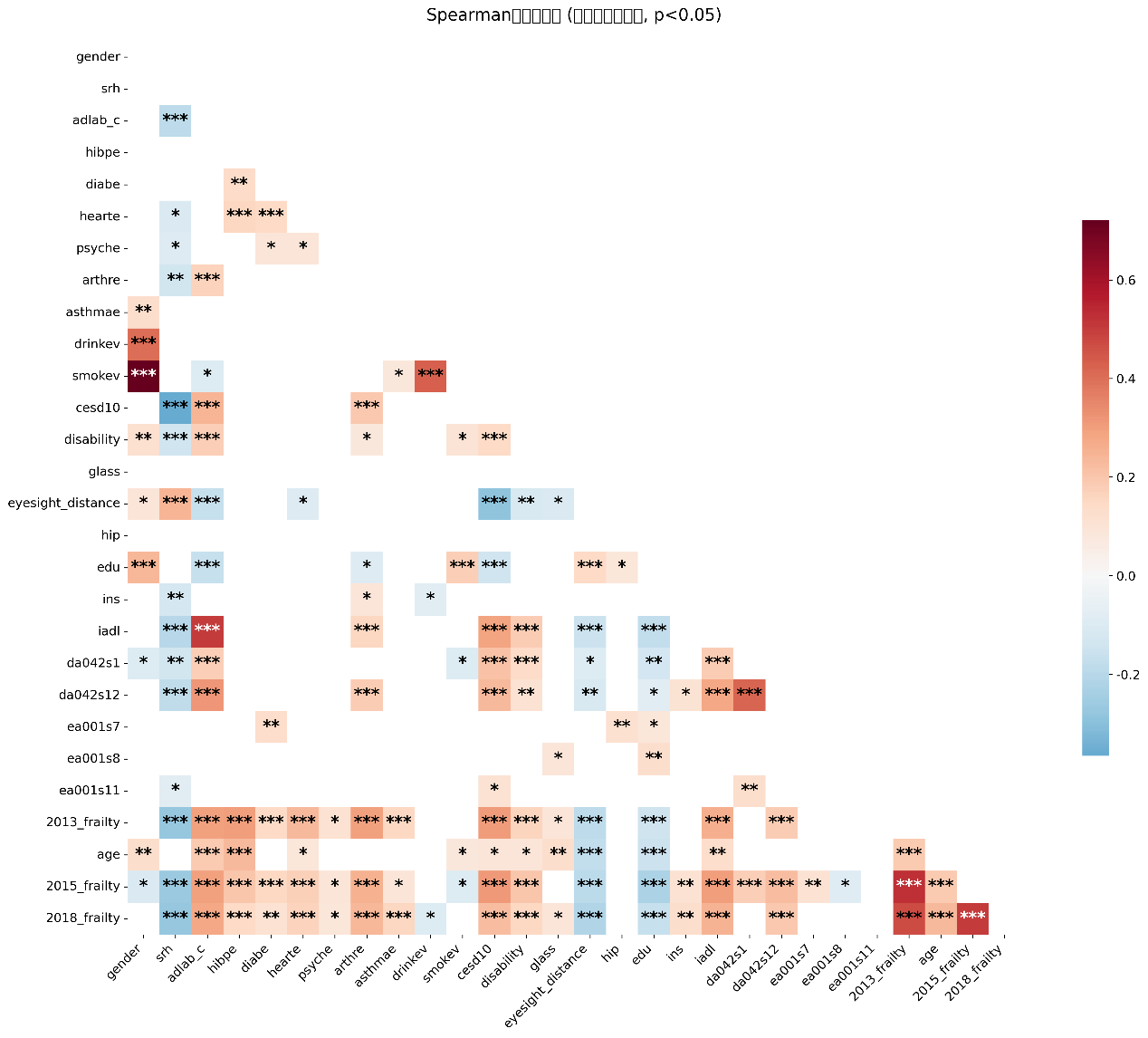
**

**Supplementary Figure 18 Spearman Correlation Significant Only**
